# Direct Reductive Amination of Carbonyl Compounds Catalyzed by a Moisture Tolerant Tin(IV) Lewis Acid

**DOI:** 10.1002/adsc.201701418

**Published:** 2018-01-15

**Authors:** Joshua S. Sapsford, Daniel J. Scott, Nathan J. Allcock, Matthew J. Fuchter, Christopher J. Tighe, Andrew E. Ashley

**Affiliations:** ^1^ Department of Chemistry Imperial College London London SW7 2AZ UK; ^2^ Department of Chemical Engineering Imperial College London London SW7 2AZ UK

**Keywords:** ‘frustrated Lewis pairs’, catalytic hydrogenation, water tolerance, reductive amination, tin

## Abstract

Despite the ever‐broadening applications of main‐group ‘frustrated Lewis pair’ (FLP) chemistry to both new and established reactions, their typical intolerance of water, especially at elevated temperatures (>100 °C), represents a key barrier to their mainstream adoption. Herein we report that FLPs based on the Lewis acid ^i^Pr_3_SnOTf are moisture tolerant in the presence of moderately strong nitrogenous bases, even under high temperature regimes, allowing them to operate as simple and effective catalysts for the reductive amination of organic carbonyls, including for challenging bulky amine and carbonyl substrate partners.

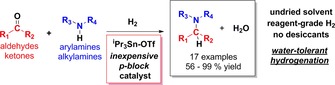

Hydrogenations catalyzed by main‐group ‘frustrated Lewis pairs’ (FLPs)^1^ have attracted enormous recent interest as potential alternatives to the use of scarce, toxic, and expensive precious transition metal (TM) catalysts. FLPs consist of Lewis acid (LA) and Lewis base (LB) pairs which are sterically precluded from irreversibly forming strong classical adducts leading to unquenched reactivity that can be utilised for bond activation processes. In particular, heterolytic cleavage of H_2_ into protic [LB−H]^+^ and hydridic [LA−H]^−^ components can be achieved and utilized for the polar hydrogenation of various substrates. Early catalytic hydrogenation protocols based on this reactivity^2^ were established for a variety of unsaturated organic functional groups containing C=N and C=C bonds, almost exclusively using organoboron‐based LA catalysts, typified by B(C_6_F_5_)_3_. Though they have provided a dramatic proof‐of‐principle for TM‐free catalytic hydrogenation, such systems suffer from a number of common limitations. In particular, and in the vast majority of cases, H_2_O (and other compounds containing the hydroxyl group) is a potent catalyst poison, forming highly Brønsted acidic adducts with the LA [e. g. H_2_O⋅B(C_6_F_5_)_3_: p*K*_a_=8.4 (MeCN), <1 (aq., *est*.), similar to HCl].^3^ Such adducts can be irreversibly deprotonated by even moderately strong bases (e. g., alkyl imines/amines) to the corresponding oxyborate anions, which are catalytically inactive (Scheme 1). Furthermore, susceptibility to decomposition *via* B−C protonolysis at relatively modest temperatures (>100 °C)^4^ means that reversibility cannot be imparted through heating (which also restricts the upper operating temperature, narrowing the opportunity to optimise rates of conversion).

**Scheme 1:**
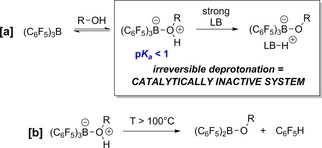
Detrimental effects of hydroxylic species upon catalytic activity of B(C_6_F_5_)_3_. R=alkyl, H; LB=Lewis base. [a] Brønsted acidification of H_2_O *via* coordination to B(C_6_F_5_)_3_. [b] Thermally induced protodeboronation. Quoted p*K*_a_ relates to aqueous conditions (est.).^3^

Nevertheless, in recent years we,^5^ and others,^6^ have separately reported the development of borane‐based protocols for the catalytic hydrogenation of organic carbonyls, that are tolerant of H_2_O and alcohol products. Notably, however, in none of these cases was moisture tolerance reported in the presence of basic functional groups (e. g. imines/amines), which is consistent with the need to avoid deprotonation of H_2_O⋅LA, as discussed above. This of course presents a serious drawback in terms of reaction scope. For example, the reductive amination (RA) of organic carbonyls is a powerful and versatile C−N bond forming methodology that is a key route to secondary and tertiary amines in many industrially‐important compounds; it has been reported that 20% of target drugs in leading pharmaceutical companies incorporate a RA step.^7^ While various stoichiometric reductants have been incorporated into these reactions, from an atom economy perspective *direct* RA using H_2_ as the reductant is especially attractive.

Homogeneous catalysts for RA typically use precious TMs (e. g. Ru, Rh, Ir),^8^ although a handful of non‐precious TM catalysts based on Fe or Cu have been disclosed, all of which require high pressures, anhydrous solvents and/or desiccants to perform well.^9^ In the quest for non‐precious metal RA catalyst candidates, main‐group FLP systems seem particularly appealing, given the status of imines as the ‘archetypal’ FLP hydrogenation substrate. However, successful RA necessarily requires H_2_O tolerance in the presence of imine/amine bases.^10^ Very recently Soós *et al*. reported the first example of FLP‐catalysed RA (Scheme 2)^11^ employing a triarylborane as LA (**I** in Scheme 2), which is impressive given the factors outlined above. The authors noted, however, that *“electronic tuning [in BAr_3_ species] has reached its limit”* for refining moisture tolerance, and their success was based upon very careful and specific design of the triarylborane used, which focused on steric modification. This has implications for reaction scope, which is known to be highly dependent on LA structure.2g For example, Soós’ borane design included the use of very high steric bulk, even by FLP standards; consequently, the reduction of bulky substrates was found to be especially challenging. Thus, alternative and complementary approaches to FLP‐catalysed RA are still desirable.

**Scheme 2:**
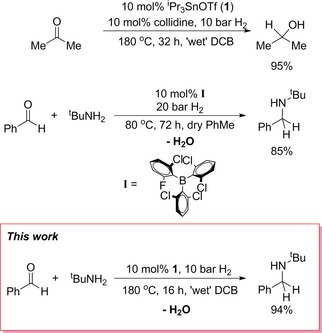
Examples of previous moisture‐tolerant FLP hydrogenation systems relevant to this work.

We have recently adopted a different approach to achieving ROH tolerance by switching to LAs based on ‘softer’ *p*‐block elements than B, and reported that inexpensive and readily‐synthesised ^i^Pr_3_SnOTf (**1**; Tf=SO_2_CF_3_) is a versatile catalyst for the FLP‐type hydrogenation of C=N, C=O and C=C bonds (Scheme 2).^12^ We also briefly noted that this LA showed appreciable moisture tolerance for the hydrogenation of acetone. Herein we extend our initial study and demonstrate that ^i^Pr_3_SnOTf is an effective RA catalyst for both aryl and alkyl amine substrates with either aldehyde or ketone coupling partners, using technical grade solvents and reagents (i. e. ‘wet’ conditions), and without the need for desiccants.^13^

Initially, we applied our protocol for carbonyl hydrogenation with **1** under ‘wet’ conditions [10 bar H_2_ (undried), reagent grade 1,2‐dichlorobenzene (DCB)] to archetypal imines PhC(H)=NPh (**2 a**, Scheme 3) and PhC(H)=N^t^Bu (**2 b**, Scheme 3). While turnover can be successfully achieved at 120 °C for these substrates under anhydrous conditions,^12^ when moisture is present the temperature must be raised to 180 °C to overcome its inhibitory effect, yet this is made possible by the thermally robust nature of **1**. Perhaps unexpectedly, the use of either molecular sieves (3 or 4 Å) or anhydrous MgSO_4_ as desiccants proved to be deleterious to the reaction rate, which we similarly ascribe to the competitive adsorption of the Sn catalyst to the surface oxygen sites of these materials.6d As when employing anhydrous conditions, collidine (2,4,6‐trimethylpyridine, Col; p*K_a_*=7.4 in H_2_O)^14^ was required as an auxiliary base only for **2 a**, which is too weakly basic to activate H_2_ directly with **1** at a feasible rate; conversely the higher basicity of **2 b** allows the imine and product amine (p*K_a_* 10.5 in H_2_O)^15^ to act as the LBs for H_2_ cleavage. In this latter case, however, the enhanced basicity also leads to a requirement for longer reaction times, which we ascribe to increased deprotonation of the aqua species [^i^Pr_3_Sn⋅2H_2_O]^+^ (p*K*_a_=6.37 in aqueous EtOH)^16^ to off‐cycle ^i^Pr_3_SnOH/(^i^Pr_3_Sn)_2_O, thus reducing the concentration of the active LA catalyst. Encouragingly, despite observing partial hydrolysis of **2 a**/**2 b** to PhCHO and PhNH_2_/^t^BuNH_2_ immediately upon dissolving at RT (by ^1^H NMR; see SI), only *ca*. 5% of the side‐product PhCH_2_OH was detected at the end of these reactions.

**Scheme 3:**
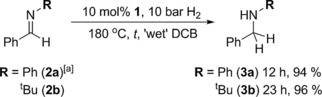
^i^Pr_3_SnOTf‐catalysed hydrogenation of imines under ‘wet’ conditions. [a] 10 mol% Col added. 10 bar refers to initial pressure at RT. All reactions were prepared on the open bench and degassed before pressurisation. Percentages are *in situ* conversions determined by ^1^H NMR spectroscopy (see SI for full details).

Based on these successful initial results, we attempted the RA of PhCHO and PhNH_2_, as a model reaction (Table 1, entry **3 a**). Upon mixing these substrates with no catalyst, 31% conversion to imine **2 a** was observed by ^1^H NMR spectroscopy, over 24 hours. Subsequent addition of **1**, however, resulted in immediate further conversion to **2 a** (87%), concomitant with a visible phase separation between the DCB solvent and H_2_O generated from the condensation reaction; evidently, **1** acts as an efficient LA catalyst to promote imine formation from carbonyls and amines. Gratifyingly, the conditions used for ‘wet’ imine hydrogenation were applicable to the RA, with an excellent conversion of 94% to the target amine; the exclusive side‐product was PhCH_2_OH. A longer reaction time for the RA was required than that for the direct hydrogenation of imine **2 a** under ‘wet’ conditions, which is to be expected from the greater amount of H_2_O present, formed from the initial condensation reaction. We propose that the reduction mechanism is likely to be the same as that proposed for imine hydrogenation with **1**, in which H_2_ activation by **1/**Col precedes protonation of **2 a** by [Col−H]^+^[OTf]^−^, prior to subsequent reduction of the [**2 a**‐H]^+^[OTf]^−^ to **3 a** by ^i^Pr_3_Sn−H (regenerating **1**), all *via* a polar mechanism.^12^ Here, the effect of H_2_O is to bind to **1** and reversibly sequester it as off‐cycle species (*vide supra*), thereby retarding the rate of H_2_ activation.^17^ Attempts to lower the catalyst loading to 5 mol% led to a dramatic drop in rate; given that no evidence of appreciable decomposition was observed, this is attributed simply to a doubling of the H_2_O/catalyst ratio. Additionally, changing the solvent to toluene detrimentally affected the reaction rate, primarily due to the poor solubility of **1** in non‐polar solvents.^12^

**Table 1:** ^i^Pr_3_SnOTf‐catalysed hydrogenation of imines under ‘wet’ conditions.^[a]^

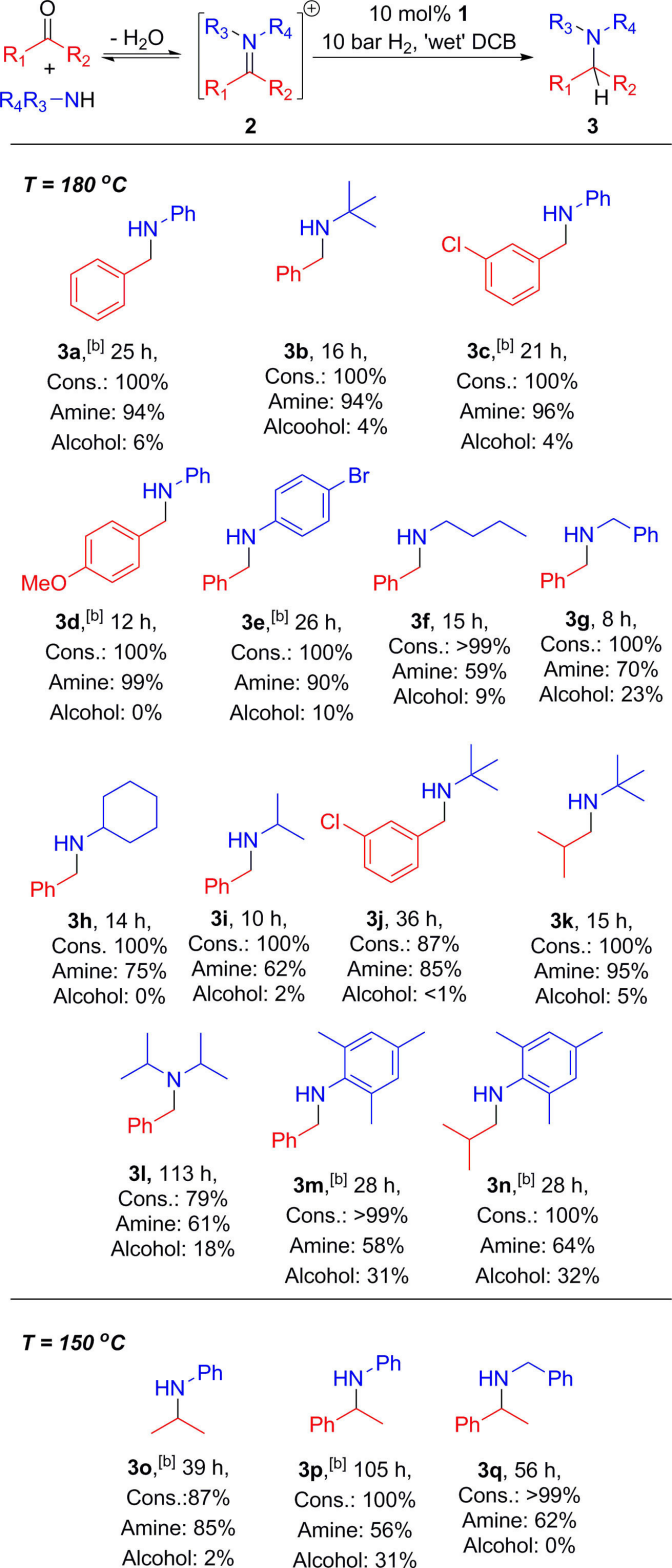

^[a]^ 10 bar refers to initial pressure at RT. All reactions were prepared on the open bench and degassed before pressurisation. Percentages are *in situ* conversions determined by ^1^H NMR spectroscopy (see SI for full details). Cons.=consumption of carbonyl, Amine=conversion to desired target pictured amine, Alcohol=conversion of carbonyl to corresponding alcohol by direct hydrogenation. ^[b]^ 10 mol% Col added.

In addition to unsubstituted **3 a**, products bearing functional groups on either of the aryl rings could also be prepared with excellent conversions, with both electron‐withdrawing and electron‐donating groups being tolerated (Table 1, **3 c**–**3 e**). Notable exceptions are NO_2_‐substituted arenes, which resulted in very complicated mixtures and intractable products; this is presumably due to radical‐mediated reduction of ArNO_2_ by the tin hydride, as has been previously documented.^18^ Reactions employing alkylamines as reagents gave mixed results. Although the least hindered primary amine substrates formed the expected products in moderate yields (Table 1, **3 f**, **3 g**), the reactions suffered from over‐alkylation as evidenced by the formation of (PhCH_2_)_2_N−^n^Bu (from ^n^BuNH_2_) or (PhCH_2_)_2_N−R (R=H, CH_2_Ph; from PhCH_2_NH_2_) as side‐products. Interestingly, when the slightly bulkier ^i^PrNH_2_ or CyNH_2_ (Cy=cyclohexyl; Table 1, **3 h**, **3 i**) were reacted with PhCHO, the target products were formed as the major species, alongside traces of (PhCH_2_)_2_N−R (R=H, CH_2_Ph); additionally, acetone and cyclohexanone were also observed in the respective ^1^H NMR spectra, indicating some C−N bond cleavage within the ^i^Pr−N and Cy−N moieties. The formation of these carbonyl compounds likely results from a transimination reaction, which could proceed *via* **1**‐mediated β‐N H^−^ abstraction from the ^i^Pr−N and Cy−N groups in **3 h** and **3 i** respectively; the resultant iminium ions would rapidly hydrolyse to acetone or cyclohexanone,^19^ and the liberated PhCH_2_NH_2_ would undergo subsequent RA reactions with PhCHO to produce (PhCH_2_)_2_N−R (R=H, CH_2_Ph), directly analogous to the aforementioned synthesis of **3 g** (see SI, Fig. S20 for further details). It is noteworthy that parallel H^−^ abstraction reactivity has been previously documented for combinations of the ubiquitous LA in FLP chemistry, B(C_6_F_5_)_3_, and ^i^Pr_2_NH/^i^Pr_2_NEt.^20^

Both of these side reactions are attributed to the high temperatures required to achieve productive catalysis with **1** when moisture is present, which reduce selectivity.^21^ Attempts to lower the temperature to 150 °C resulted in similar product distributions accompanied by a substantial decrease in reaction rate (e. g. for **3 h**, reaction at 150 °C achieved 60% conversion to the target amine in 49 h). Nevertheless it should be emphasised that, despite these competing reactions, the desired singly‐alkylated amine was the major product for all of the above reactions. While the reaction times using **1** are mostly shorter than using Soós’ catalyst **I** for identical coupling partners (*vide supra*; Scheme 2), these side reactions were not observed by the latter, which is highly likely a result of the lower operating temperature. Since our attempts to reduce the reaction temperature with **1** detrimentally affected the rate of turnover, we considered substrates which were more problematic for **I**, namely those exhibiting a larger steric profile.^11^

Gratifyingly, the bulky amine ^t^BuNH_2_ is coupled very effectively^22^ to aromatic aldehydes and even the bulky aliphatic partner ^i^PrCHO (Table 1, **3 b**, **3 j**, **3 k**). This qualitative difference in applicability between the two systems is consistent with the lower steric bulk of ^i^Pr_3_Sn−H relative to [**I**−H]^−^, which would allow for a closer approach to even very hindered imines, thus facilitating H^−^ transfer.^23^ As well as ^t^BuNH_2_, other very bulky amines could also successfully be employed (Table 1, **3 l**–**n**). Notably the very hindered secondary amine ^i^Pr_2_NH can even be used (Table 1, **3 l**), albeit proceeding at a rather sluggish rate; the side‐product profile in this reaction mirrors that from the synthesis of **3 i**, indicating a general propensity for ^i^Pr‐substituted amines to undergo RA‐transimination reactions under these conditions. The relatively high production of PhCH_2_OH is attributed to a slow initial condensation reaction (observed in the ^1^H NMR), which leaves a greater amount of PhCHO to compete as a hydrogenation substrate.

Initial attempts to expand the carbonyl scope to ketones led to a significant drop in chemoselectivity for hydrogenation, with substrates PhNH_2_ and CH_3_COCH_3_ or PhCOCH_3_ yielding ∼1:1 ratios of the target amine and the alcohol side‐product. Fortunately, and in contrast to the findings for aldehydes, for ketone substrates this selectivity *is* improved by reducing the reaction temperature to 150 °C, albeit at the cost of reduced reaction rate. Accordingly, under otherwise identical conditions, both acetone and acetophenone could be successfully coupled (Table 1, **3 o**–**q**).

Finally, in order to demonstrate the ability of **1**‐catalysed RA to produce larger quantities of material, the hydrogenative coupling of model substrates PhNH_2_ and PhCHO was conducted on an increased scale. Conducting the reaction at 150 °C and using slightly modified conditions (DCB was replaced with 1,2‐difluorobenzene to facilitate solvent removal during workup; an increased pressure of 50 bar was used to compensate for the lower reaction temperature), the reaction furnished target **3 a** with an isolated yield of 75% (343 mg; Scheme 4), following a simple work‐up.

**Scheme 4:**
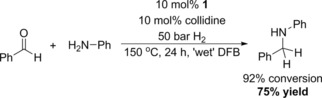
Scaled‐up reductive amination of benzaldehyde with aniline catalysed by **1**. 50 bar refers to final pressure at 150 °C (35 bar at RT). The reaction was prepared on the open bench and sparged with N_2_ before pressurisation with H_2_ (see SI). The *in situ* conversion was determined by ^1^H NMR spectroscopic analysis (see SI), while the yield was calculated from the mass of isolated pure product.

In conclusion, we have developed a simple and practical FLP‐type protocol for the RA of various amines and organic carbonyls, catalysed by ^i^Pr_3_SnOTf (**1**). This simple ‘R_3_Sn^+^’‐based Lewis acid, which can be readily prepared from inexpensive starting materials, displays a remarkable tolerance to H_2_O, elevated temperatures and strong amine bases. Notably, this protocol shows a qualitative substrate scope that is complementary to that of the only other reported FLP RA catalytic system, which was recently reported by Soós *et al*. and employed sterically‐tuned triarylboranes. Since our approach to develop a H_2_O‐tolerant LA (required for RA) manipulated electronic factors (i. e. incorporating a softer *p*‐block element), rather than augmenting the sterics of an existing LA series, the lower steric profile of **1** enables the successful reduction of more hindered substrates while still being competent for H_2_ activation, in the presence of moisture. In addition to the inherent appeal of developing new methods for precious metal‐free RA, we would also suggest that these results further emphasise the value of pursuing ‘alternative’ non‐boron‐based LAs as targets for FLP chemistry.

## **Experimental Section**

All reactions were prepared on the open bench unless stated otherwise. ^i^Pr_3_SnOTf (**1**) was synthesised according to literature.^12^ All substrates, 2,4,6‐collidine and solvents (1,2‐dichlorobenzene (DCB), 1,2‐difluorobenzene (DFB)) were purchased from commercial suppliers (Sigma Aldrich, Fluorochem, Acros Organics). Solid imines were dried under vacuum and stored under N_2_, while liquid imines and aldehydes were degassed, dried over 4 Å molecular sieves and stored under N_2_. All other compounds and solvents were used as supplied. H_2_ was purchased from BOC (research grade) and used without further drying or purification.

### Typical Procedure for the ‘Open Bench’ Hydrogenation of Imines Catalysed by 1

To a solution of imine (0.2 mmol) (and, for imine **2 a** only, 2,4,6‐collidine (2.6 μL, 0.02 mmol, 10 mol%)) in 1,2‐dichlorobenzene (0.7 mL) was added to 1 (7.9 mg, 0.02 mmol, 10 mol%) in a Wilmad high pressure NMR tube fitted with a PV‐ANV PTFE valve. The solution was freeze‐pump‐thaw degassed once. After complete thawing, H_2_ was admitted up to a pressure of 10 bar at RT. The reaction mixture was heated in an Al bead bath; the results are presented in Scheme 3.

### Typical Procedure for the ‘Open Bench’ Hydrogenation Aminations Catalysed by 1

To a solution of amine (0.2 mmol), carbonyl (0.2 mmol) and, when aniline or its derivatives are used (e. g. **3 a**, **3 g**), 2,4,6‐collidine (2.6 μL, 0.02 mmol, 10 mol%) in 1,2‐dichlorobenzene (0.7 mL) was added to 1 (7.9 mg, 0.02 mmol, 10 mol%) in a Wilmad high pressure NMR tube fitted with a PV‐ANV PTFE valve. The solution was freeze‐pump‐thaw degassed once. After complete thawing, H_2_ was admitted up to a pressure of 10 bar at RT. The reaction mixture was heated in an Al bead bath, and the results are presented in Table 1.

### Procedure and for the Scaled‐up Reductive Amination Catalysed of PhCHO and PhNH_2_ Catalysed by 1

A solution of 1 (99.3 mg, 0.25 mmol) in 1,2‐difluorobenzene (35 mL) was prepared in a 100 mL Parr 5500 high pressure compact laboratory reactor. The reactor was sealed and sparged with N_2_ for 5 minutes, then pressurised with nitrogen (10 bar) and stirred for a further 5 minutes. The reactor was depressurised, and aniline (0.228 mL, 2.50 mmol), benzaldehyde (0.254 mL, 2.50 mmol) and 2,4,6‐collidine (33.0 μL, 0.25 mmol) were injected. The reactor was pressurised with hydrogen (35.0 bar, which equates to 50 bar at 150 °C) and heated to 150 °C whilst stirring at 200 rpm. Upon completion of the reaction, the stirrer was stopped, whereupon the reactor was cooled to room temperature and depressurised. The solvent was removed under reduced pressure, resulting in a dark brown oil. The product was extracted into pentane (10 mL), where it was recrystallised by cooling to −20 °C to obtain 3a as an off‐white crystalline solid (343 mg, 1.87 mmol, 75%).

## Supporting information

As a service to our authors and readers, this journal provides supporting information supplied by the authors. Such materials are peer reviewed and may be re‐organized for online delivery, but are not copy‐edited or typeset. Technical support issues arising from supporting information (other than missing files) should be addressed to the authors.

Supplementary
