# Supplementary material for: Direct Reductive Amination of Carbonyl Compounds Catalyzed by a Moisture Tolerant Tin(IV) Lewis Acid
Source: Adv Synth Catal. 2018 Jan 15;360(6):1066–71. doi: 10.1002/adsc.201701418 (PMC5901005; doi:10.1002/adsc.201701418)
Supplement: Supplementary file 1 — Supplementary [file ADSC-360-1066-s001.pdf]

Advanced Synthesis & Catalysis

Supporting Information

Supporting Information

Direct Reductive Amination of Carbonyl Compounds Catalyzed by a Moisture Tolerant Tin(IV) Lewis Acid

Joshua S. Sapsford,[†] Daniel J. Scott,[†] Nathan J. Allcock,[†] Matthew J. Fuchter,[†] Christopher J. Tighe[‡] and Andrew E. Ashley*,[†]

[†]Department of Chemistry, Imperial College London, London SW7 2AZ, UK.

[‡]Department of Chemical Engineering, Imperial College London, London SW7 2AZ, UK.

Contents

1. General Experimental Considerations S3
2. Typical procedure and NMR spectra for the ‘open bench’ hydrogenation of imines catalysed by **1** S4
3. Typical procedure for ‘open bench’ reductive aminations catalysed by **1** S8
4. Mechanism for transimination S34
5. Procedure to probe the influence of water and bases on the ^{1}H and ^{119}Sn NMR shifts of $[^{i}Pr_3Sn \cdot 2(\mathit{H}_2O)]^+$ S35
6. Procedure and NMR spectra for the scaled-up reductive amination catalysed of PhCHO and $PhNH_2$ catalysed by **1** S42
7. References S45

1. General Experimental Considerations

All reactions were prepared on the open bench unless stated otherwise. $^{i}Pr_3SnOTf$ (**1**) was synthesised according to literature.[1] All substrates, 2,4,6-collidine and solvents (1,2-dichlorobenzene (DCB), 1,2-difluorobenzene (DFB)) were purchased from commercial suppliers (Sigma Aldrich, Fluorochem, Acros Organics). Solid imines were dried under vacuum and stored under N_2, while liquid imines and aldehydes were degassed, dried over 4Å molecular sieves and stored under N_2. All other compounds and solvents were used as supplied. H_2 was purchased from BOC (research grade) and used without further drying or purification. NMR spectra were recorded on Bruker AV-400 and DRX-400 spectrometers. ^{1}H NMR spectra were referenced internally to $SiMe_4$ (where applicable) or residual proteo solvent signals, while $^{119}Sn\{^{1}H\}$ NMR spectra were referenced externally to $SnMe_4$. $^{119}Sn\{^{1}H\}$ NMR spectra are only provided where resonances were observed at the start or end of the reaction. Chemical shifts are reported in ppm.

Conversions were calculated by ^{1}H NMR integration, either by relative integration of product and starting material resonances (in cases where only the starting materials and products were observed at the end-point of the reaction) or by integration relative to $SiMe_4$ (TMS) added as an internal standard. In order to minimise errors, integrations were performed on the most intense product/substrate resonances where possible, and only on signals that were well separated from other peaks. Peaks attributable to the desired product in the resulting crude product mixture have been given for each reaction.

2. Typical procedure and NMR spectra for the 'open bench' hydrogenation of imines catalysed by 1

To a solution of imine (0.2 mmol) (and, for imine **2a** only, 2,4,6-collidine (2.6 µL, 0.02 mmol, 10 mol%)) in 1,2-dichlorobenzene (0.7 mL) was added to **1** (7.9 mg, 0.02 mmol, 10 mol%) in a Wilmad high pressure NMR tube fitted with a PV-ANV PTFE valve. The solution was freeze-pump-thaw degassed once. After complete thawing, H_2 was admitted up to a pressure of 10 bar at RT. The reaction mixture was heated in an Al bead bath; the results are presented in Scheme S1.

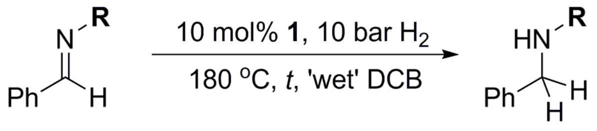

R = Ph (**2a**)[a] | **R** = Ph (**3a**) 12 h, 94 %

^{t}Bu (**2b**) | ^{t}Bu (**3b**) 23 h, 96 %

Scheme S1: 1-catalysed hydrogenation of imines under 'wet' conditions.

10 bar refers to initial pressure at RT. All reactions were prepared on the open bench and degassed before pressurisation. Percentages are *in situ* conversions determined by ^{1}H NMR spectroscopy.

[a] 10 mol% Coll added.

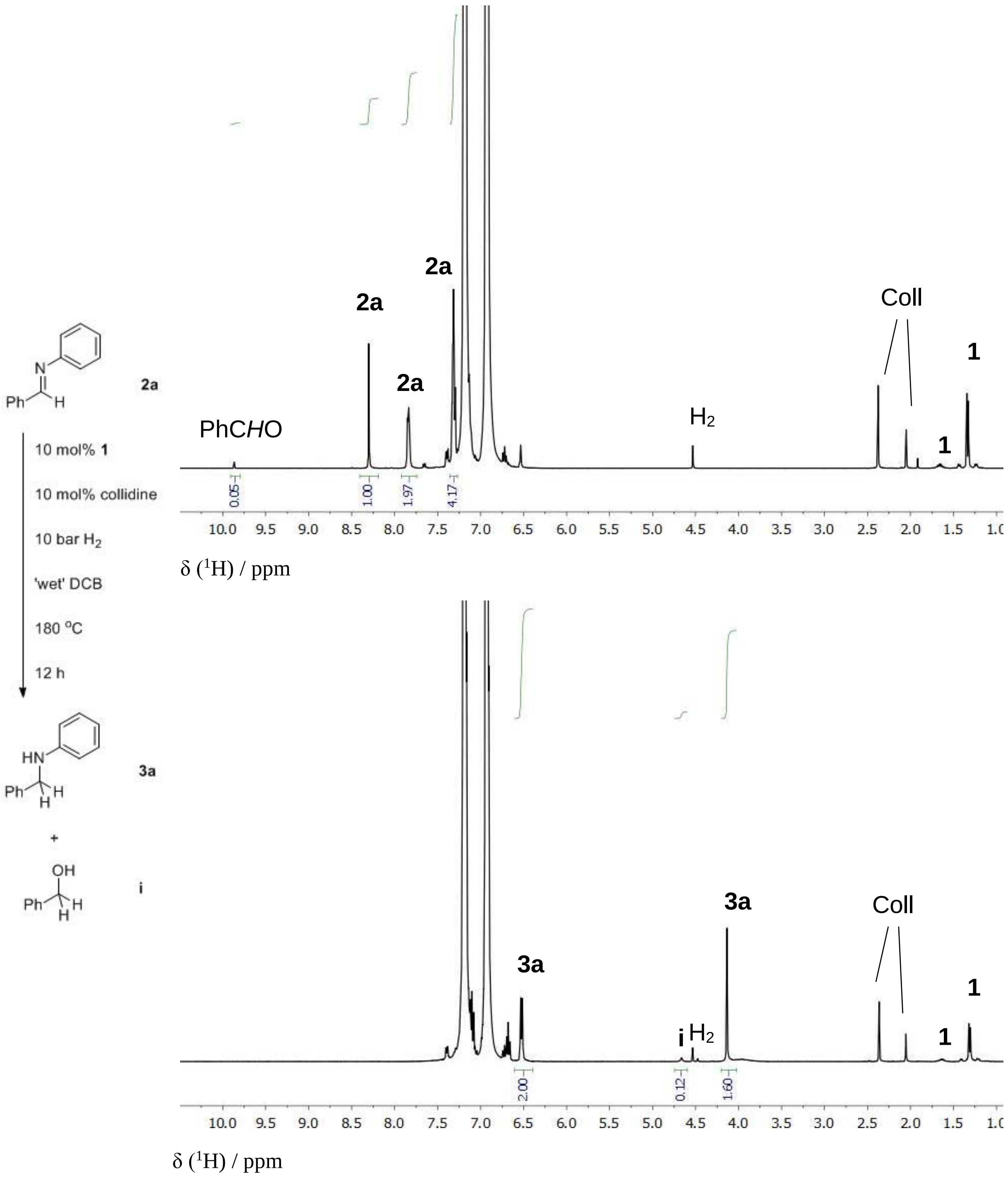

Figure S1: ^{1}H NMR spectra for the hydrogenation of **2a**. Conversion (%): **3a** (94), **i** (6). Presence of products confirmed by comparison to spectral data of pure, authentic compounds: **3a**,[1] **i**.[2] PhCHO is formed from hydrolysis of **2a**.

3a ^{1}H NMR (400 MHz): δ = 6.52 (d, $^3J_{HH}$ = 7.8 Hz), 2H, pyridyl Ar–*H*), 4.14 (s, 2H, N–CH_2), 3.96 (br s, 1H, N*H*).

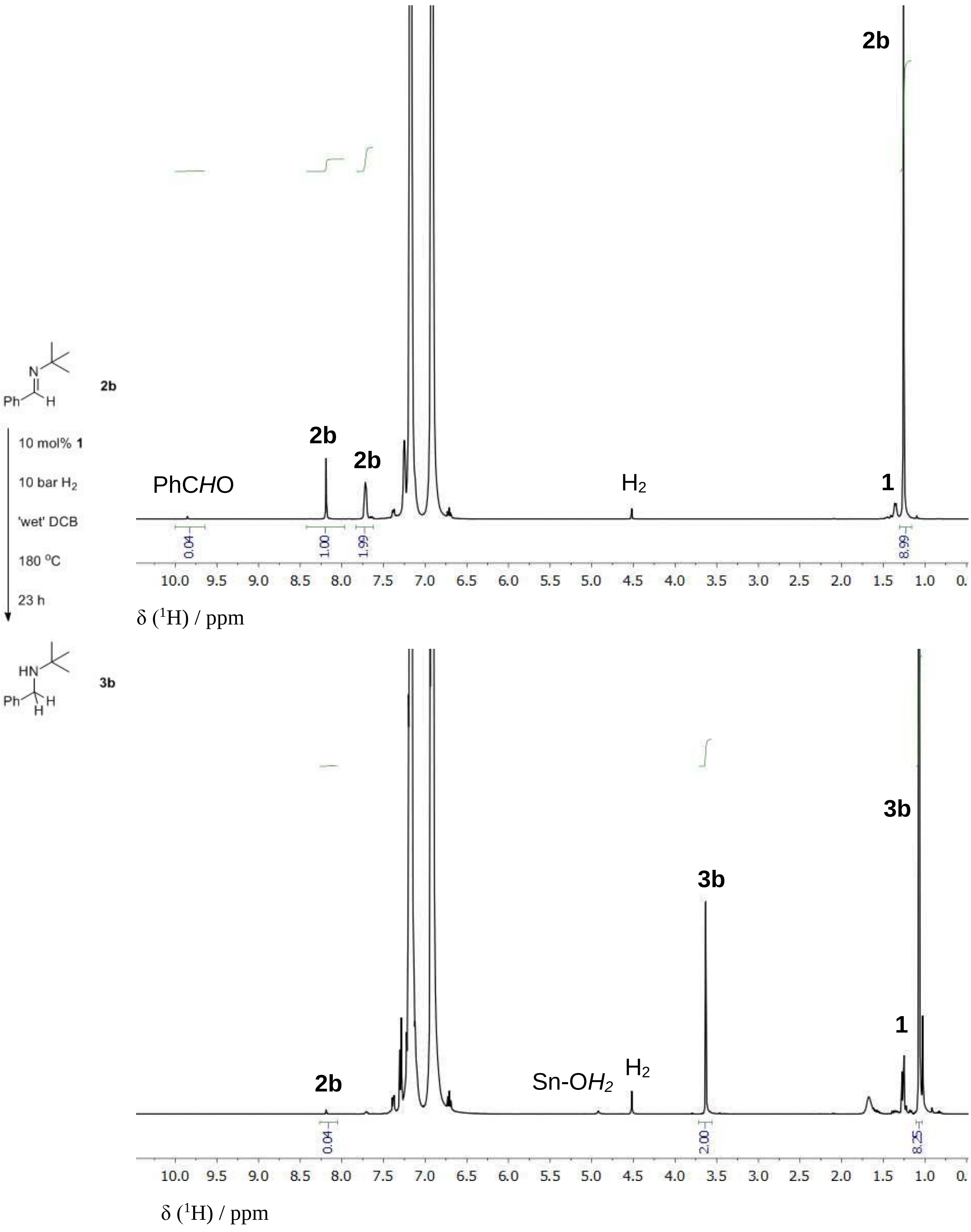

Figure S2a: ^{1}H NMR spectra for the hydrogenation of **2b**. Conversion (%): **3b** (96). Presence of products confirmed by comparison to spectral data of pure, authentic compounds: **3b**.[3] PhCHO is formed from hydrolysis of **2b**.

3b ^{1}H NMR (400 MHz): δ = 3.63 (s, 2H, N–CH_2), 1.07 (s, 9H, C(CH_3)$_3$).

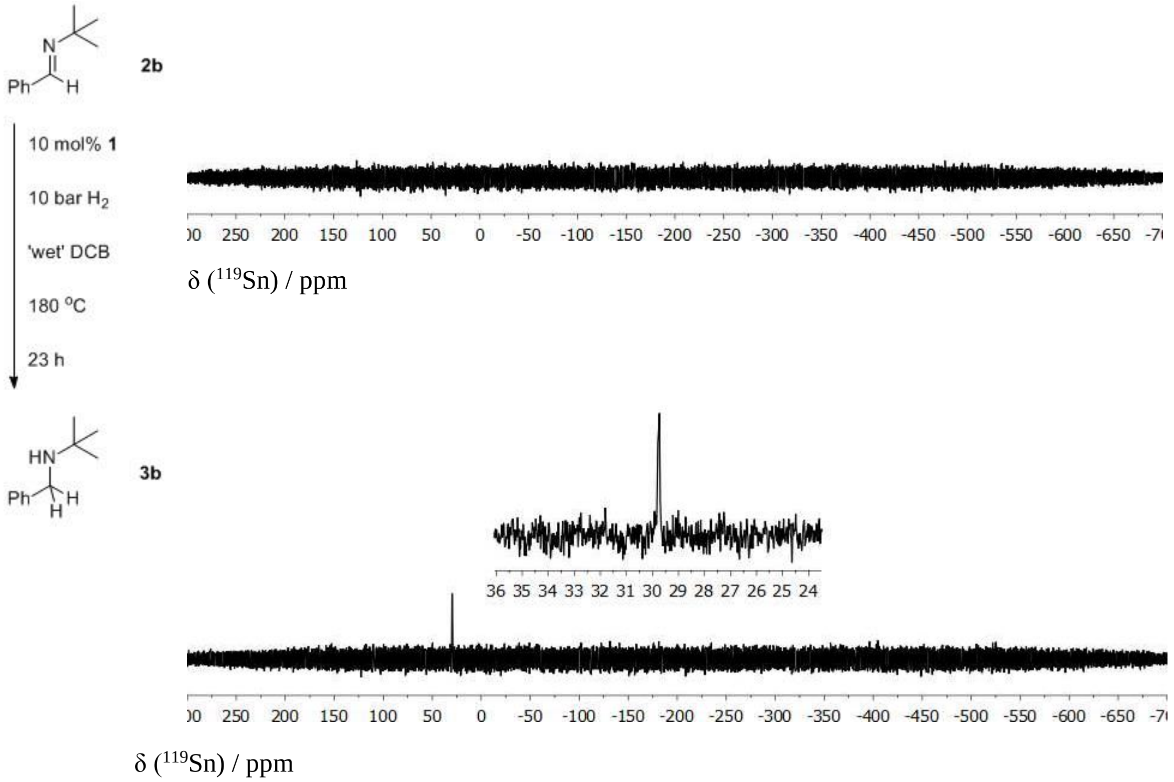

Figure S2b: $^{119}Sn\{^{1}H\}$ NMR spectra for the hydrogenation of **2b**. Inset provides expanded view of the observed resonance.

3. Typical procedure for 'open bench' reductive aminations catalysed by 1

To a solution of amine (0.2 mmol), carbonyl (0.2 mmol) and, when aniline or its derivatives are used (e.g. **3a**, **3g**), 2,4,6-collidine (2.6 µL, 0.02 mmol, 10 mol%) in 1,2-dichlorobenzene (0.7 mL) was added to **1** (7.9 mg, 0.02 mmol, 10 mol%) in a Wilmad high pressure NMR tube fitted with a PV-ANV PTFE valve. The solution was freeze-pump-thaw degassed once. After complete thawing, H_2 was admitted up to a pressure of 10 bar at RT. The reaction mixture was heated in an Al bead bath, and the results are presented in Table S1.

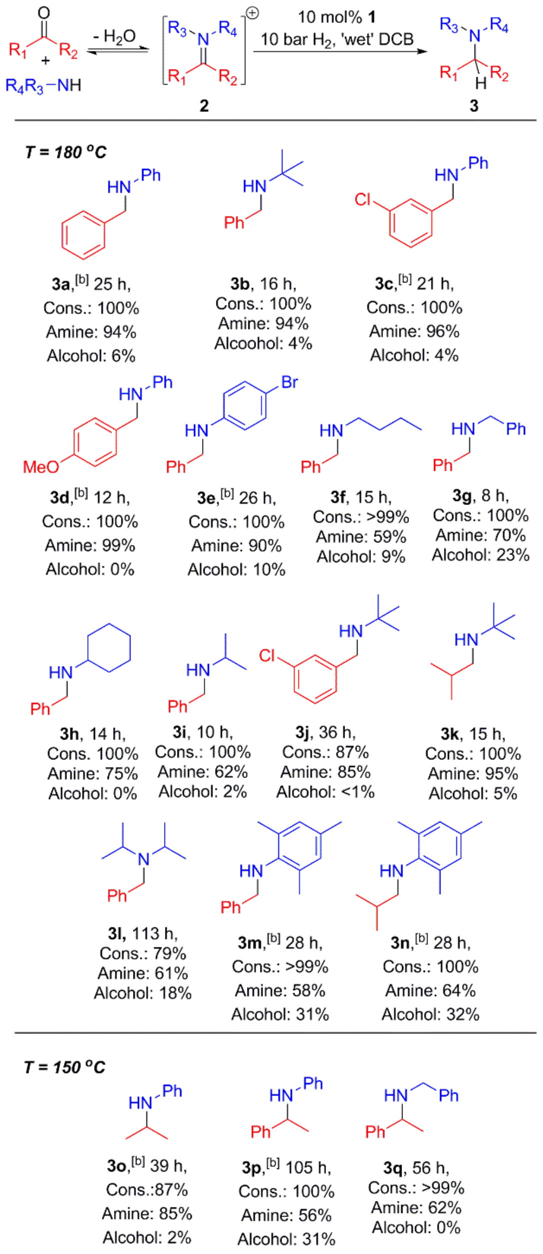

Table S1: 1-catalysed reductive amination of carbonyls and amines.

10 bar refers to initial pressure at RT. All reactions were prepared on the open bench and degassed before pressurisation. Percentages are in situ conversions determined by ^{1}H NMR spectroscopic analysis. Cons. = consumption of carbonyl, Amine = conversion to desired target pictured amine, Alcohol = conversion of carbonyl to corresponding alcohol by direct hydrogenation. [a]10 mol% Coll added.

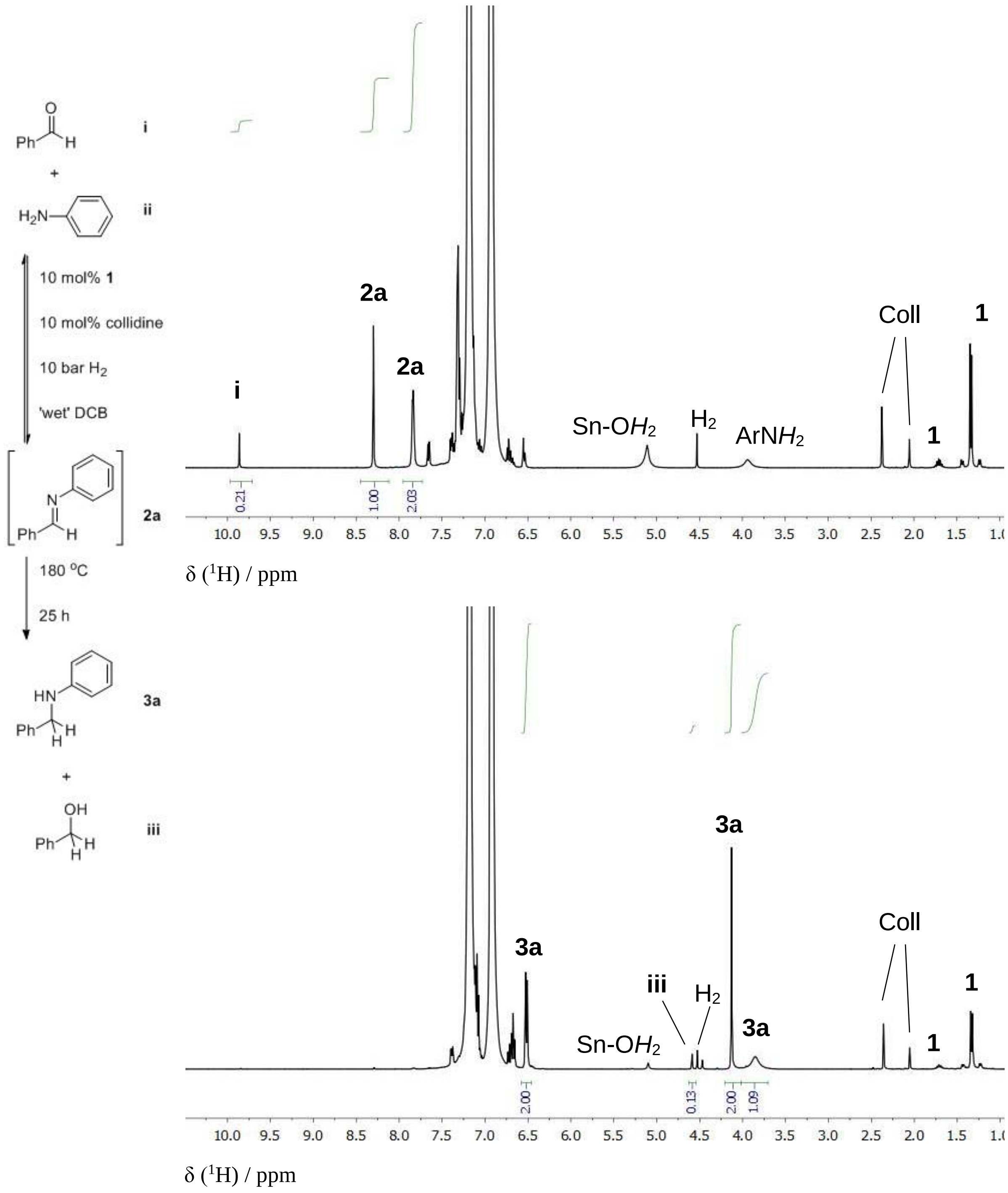

Figure S3a: ^{1}H NMR spectra for the reductive amination to **3a**. Conversion (%): **3a** (94), **iii** (6). Presence of products confirmed by comparison to spectral data of pure, authentic compounds: **2a**,[4] **3a**,[4] **iii**.[2]

3a ^{1}H NMR (400 MHz): δ = 6.52 (d, $^3J_{HH}$ = 7.8 Hz), 2H, pyridyl Ar–*H*), 4.13 (s, 2H, N–CH_2), 3.85 (br s, 1H, N*H*).

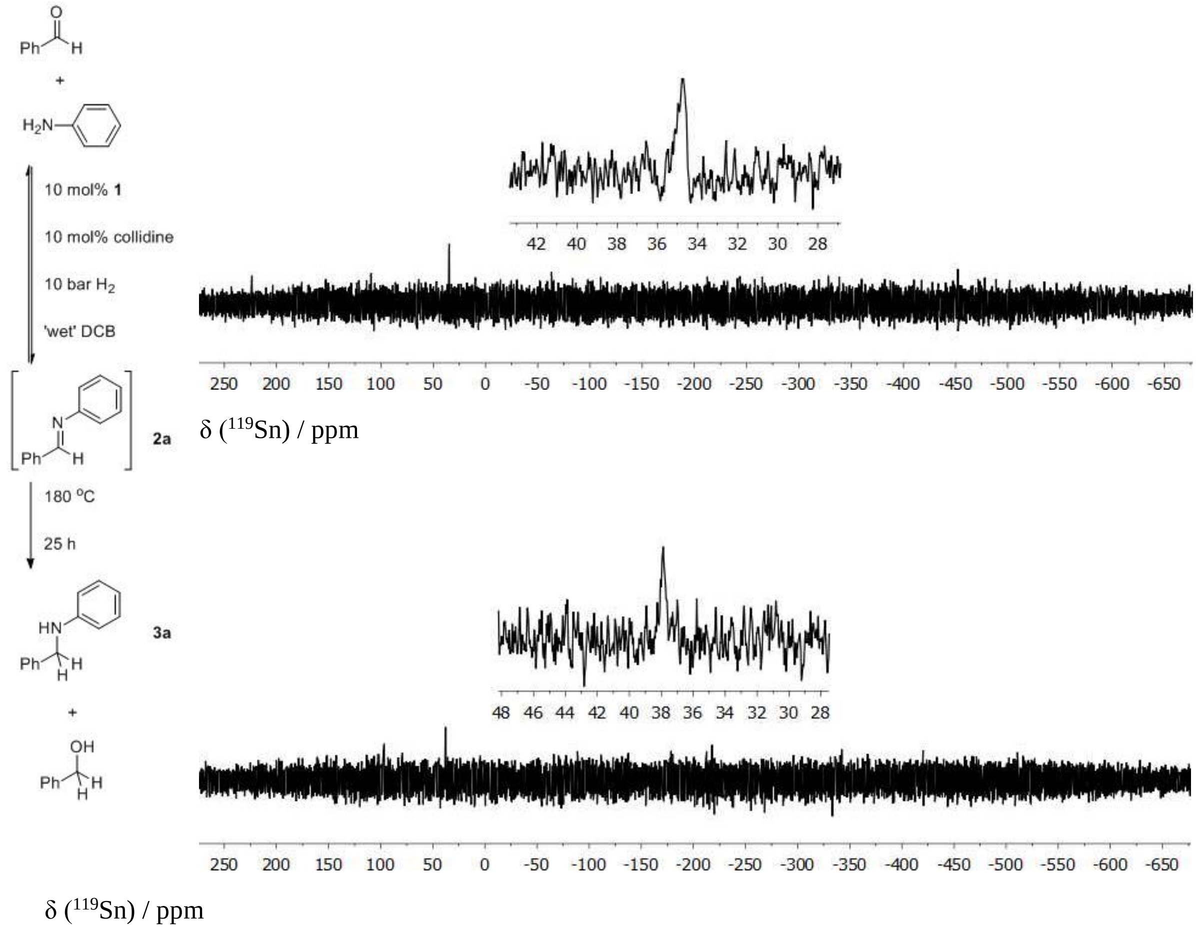

Figure S3b: $^{119}Sn\{^1H\}$ NMR spectra for the reductive amination to **3a**. Inset provides expanded view of the observed resonance.

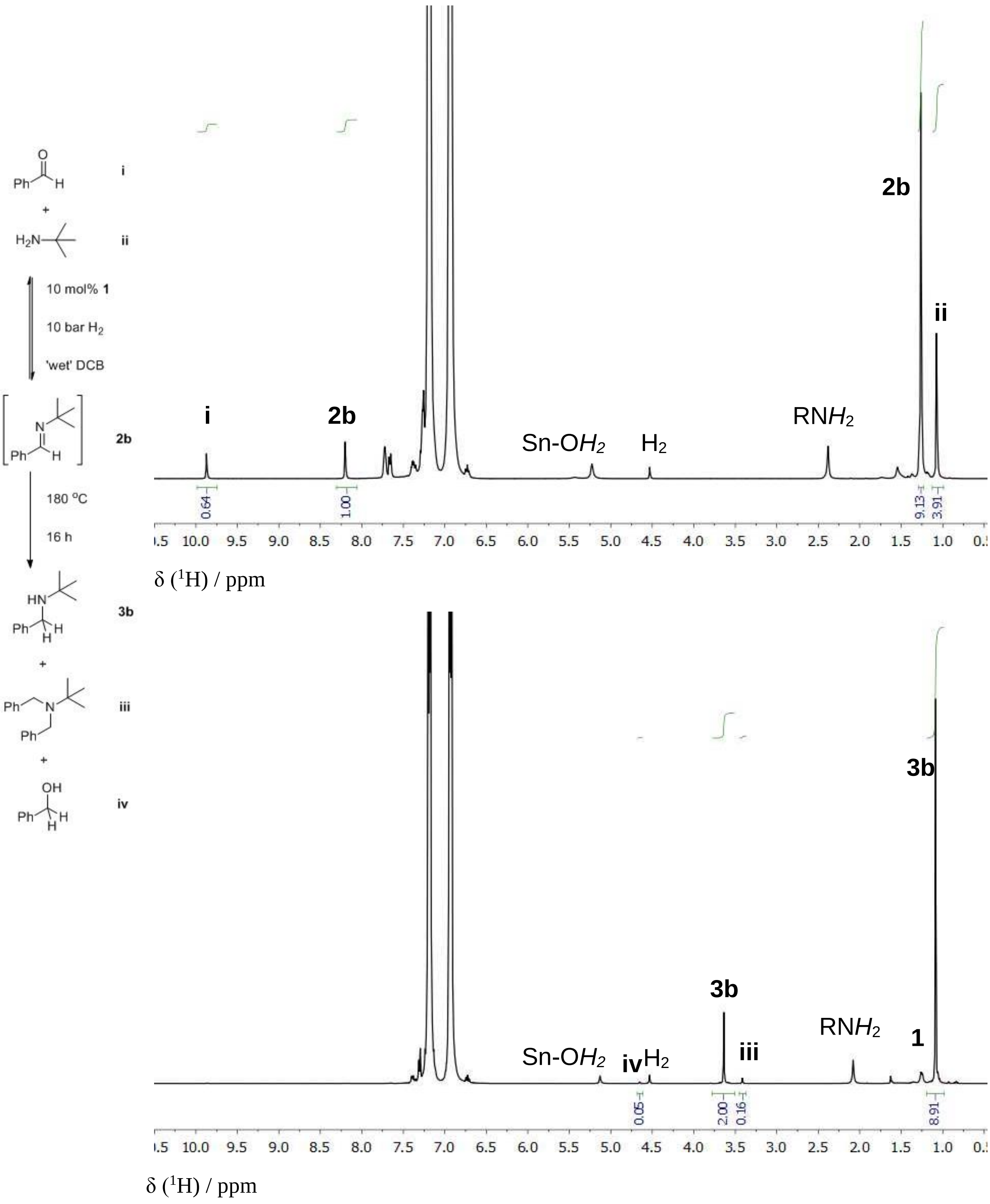

Figure S4: ^{1}H NMR spectra for the reductive amination to **3b**. Conversion (%): **3b** (94), **iii** (4), **iv** (2). Presence of products confirmed by comparison to spectral data of pure, authentic compounds: **2b**[5], **3b**[3], **iii**,[6] **iv**.[2] **iii** arises from over-alkylation (RA of **3b** and **i**).

3b ^{1}H NMR (400 MHz): δ = 3.64 (s, 2H, N–CH_2), 1.09 (s, 9H, C(CH_3)$_3$).

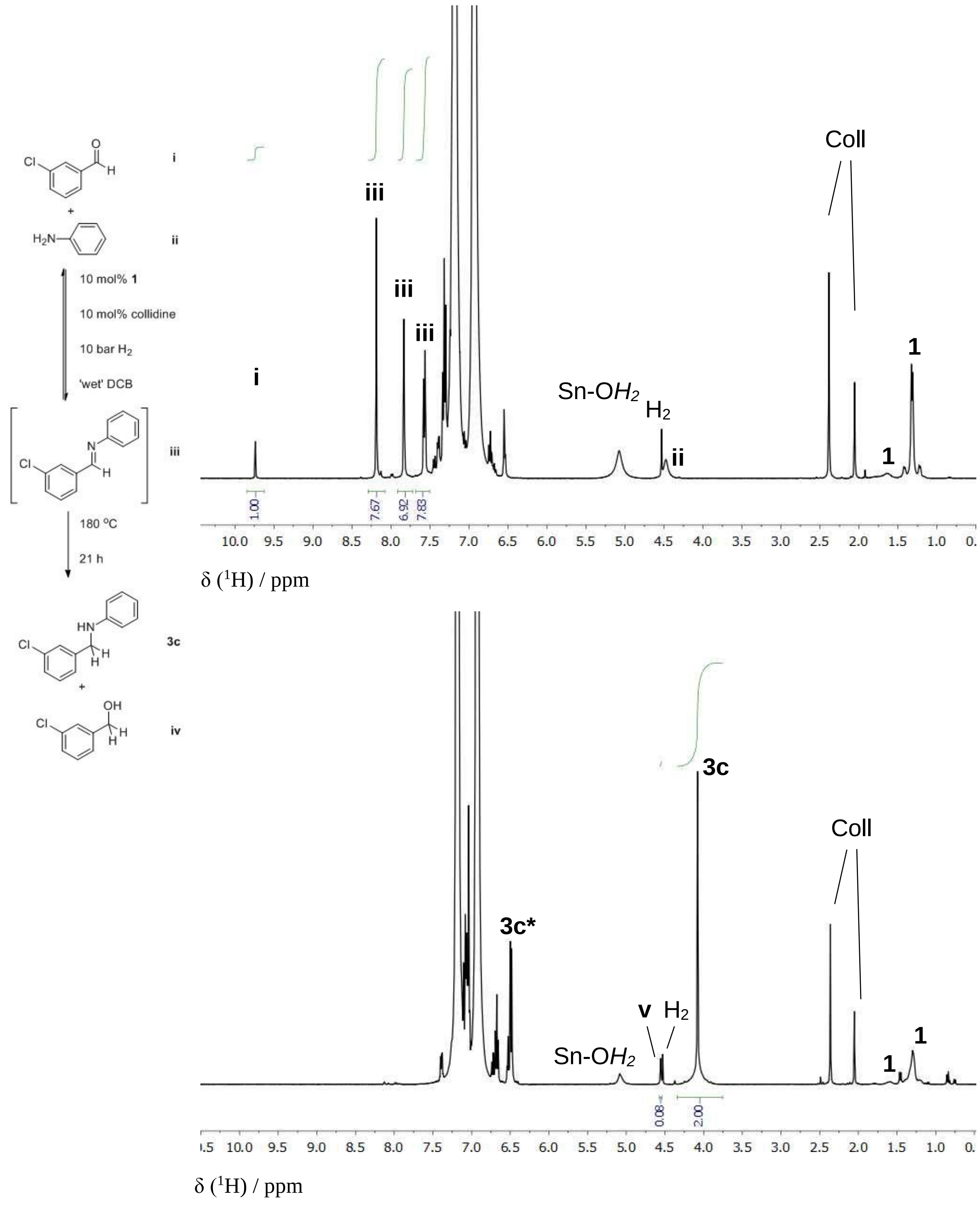

Figure S5: ^{1}H NMR spectra for the reductive amination to **3c**. Conversion (%): **3c** (96), **iv** (4). Presence of products confirmed by comparison to spectral data of pure, authentic compounds: **iii**,[7] **3c**,[8] **iv**.[9]

* **3c** aromatic peak overlaps with other aromatic peaks.

3c ^{1}H NMR (400 MHz): δ = 6.67 (t, $^{3}J_{HH}$ = 7.3 Hz), 1H, pyridyl 4-Ar–*H*), 6.49 (d, $^{3}J_{HH}$ = 7.8 Hz, Ar–*H*), 4.08 (s, 2H, N–C*H*$_2$).

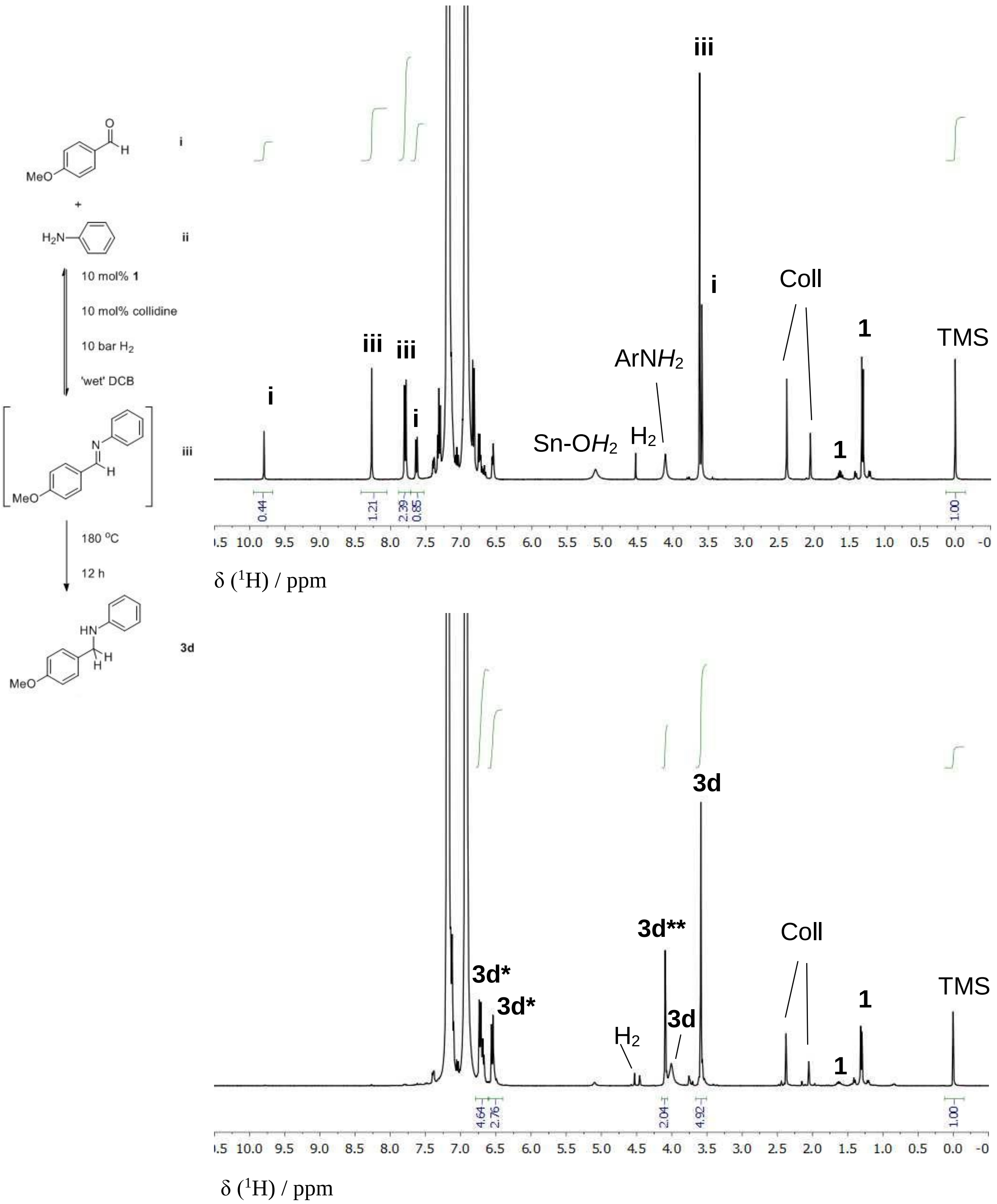

Figure S6a: ^{1}H NMR spectra for the reductive amination to **3d**. Conversion (%): **3d** (99). Presence of the product confirmed by comparison to spectral data of pure, authentic compound: **3d**.[10]

* **3d** aromatic peaks overlap with other aromatic peaks.

** **3d** PhC*H*$_2$N peak overlaps with the N*H* resonance.

3d ^{1}H NMR (400 MHz): δ = 6.74-6.67 (m, 4H, Ar–*H*), 6.55 (d, $^{3}J_{HH}$ = 7.8 Hz, 2H, Ar–*H*), 4.09 (s, 2H, N–C*H*$_2$), 4.01 (br s, 1H, N*H*), 3.59 (s, 3H, Ar–OC*H*$_3$).

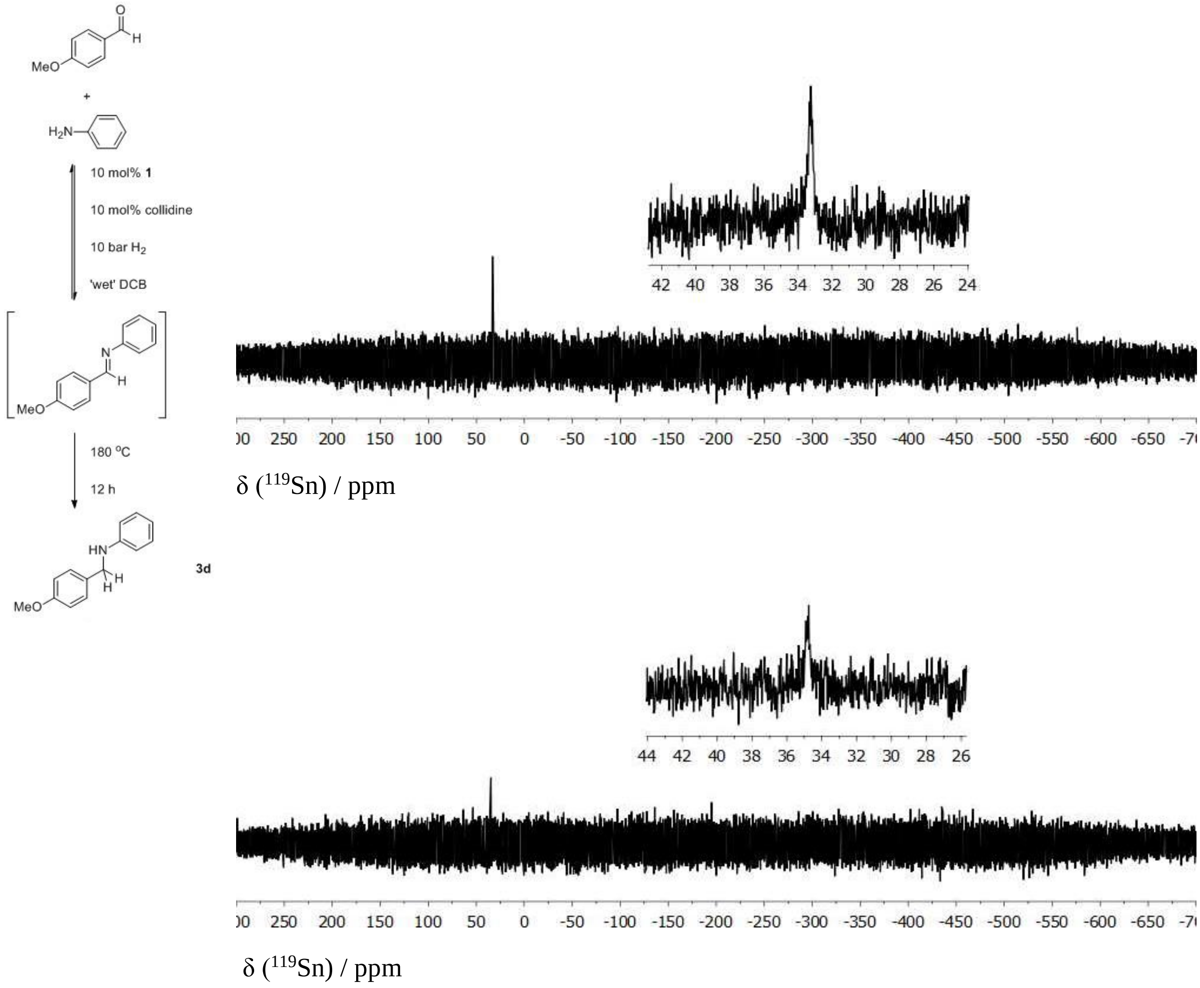

Figure S6b: $^{119}Sn\{^1H\}$ NMR spectra for the reductive amination to **3d**. Inset provides expanded view of the observed resonance.

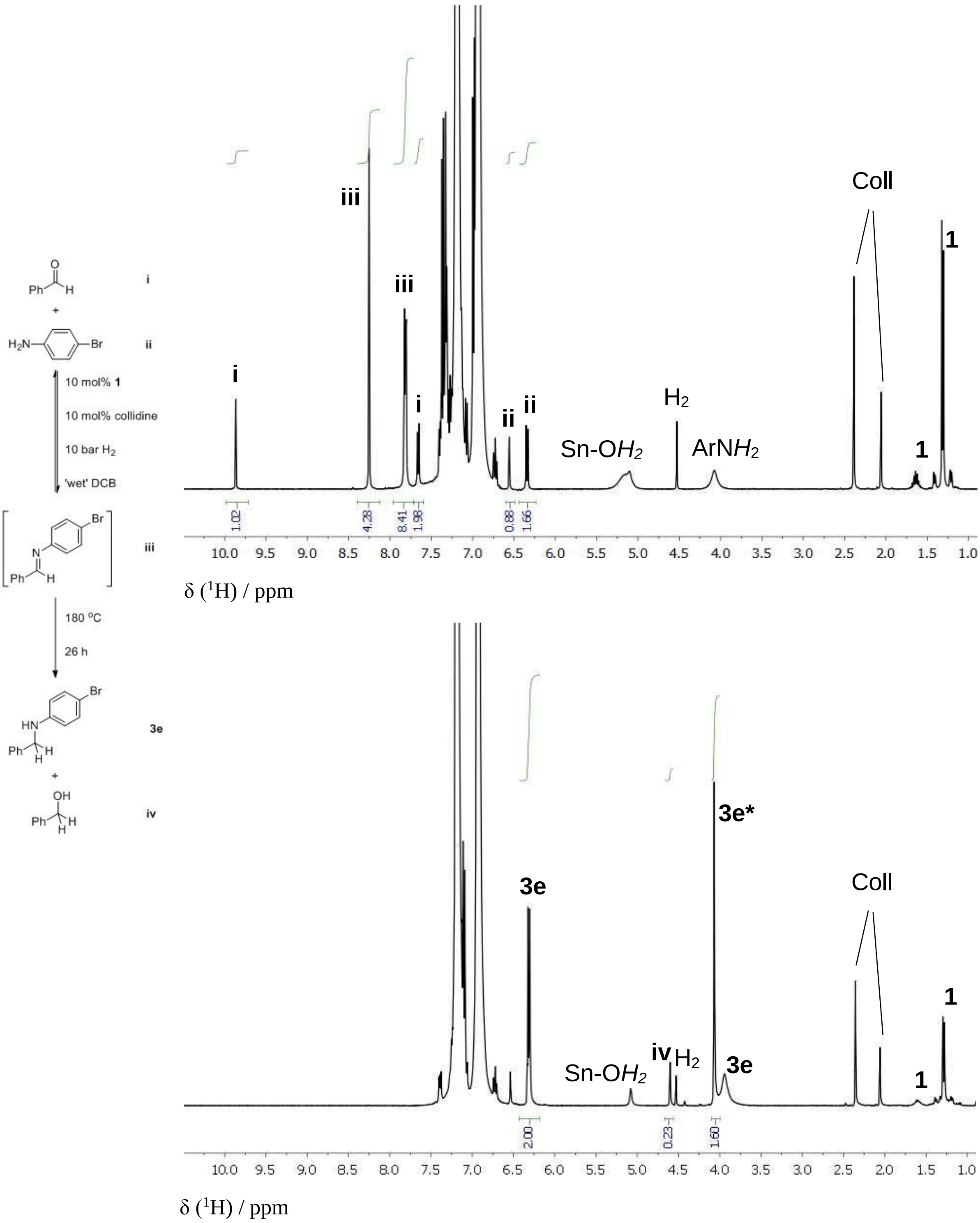

Figure S7a: ^{1}H NMR spectra for the reductive amination to **3e**. Conversion (%): **3e** (90), **iv** (10). Presence of products confirmed by comparison to spectral data of pure, authentic compounds: **iii**,[11] **3e**,[12] **iv**.[2]

* **3e** PhC*H*$_2$N peak overlaps with N*H* resonance

3e ^{1}H NMR (400 MHz): δ = 6.31 (d, $^3J_{HH}$ = 8.8 Hz, 2H, Ar–*H*), 4.07 (s, 2H, N–C*H*$_2$), 3.94 (br s, 1H, N*H*).

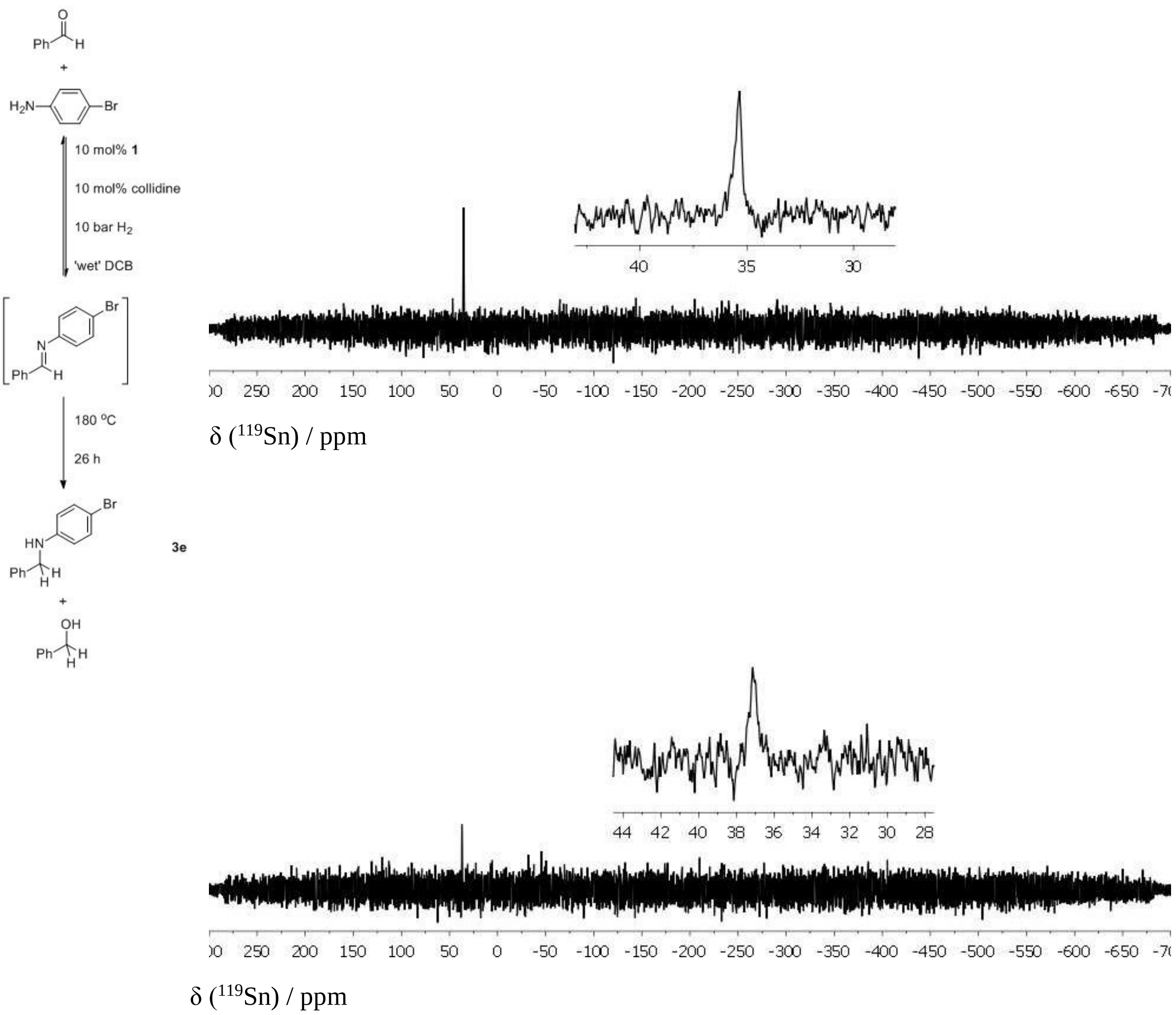

Figure S7b: ^{119}Sn {^{1}H} NMR spectra for the reductive amination to **3e**. Insets provide expanded views of the observed resonances.

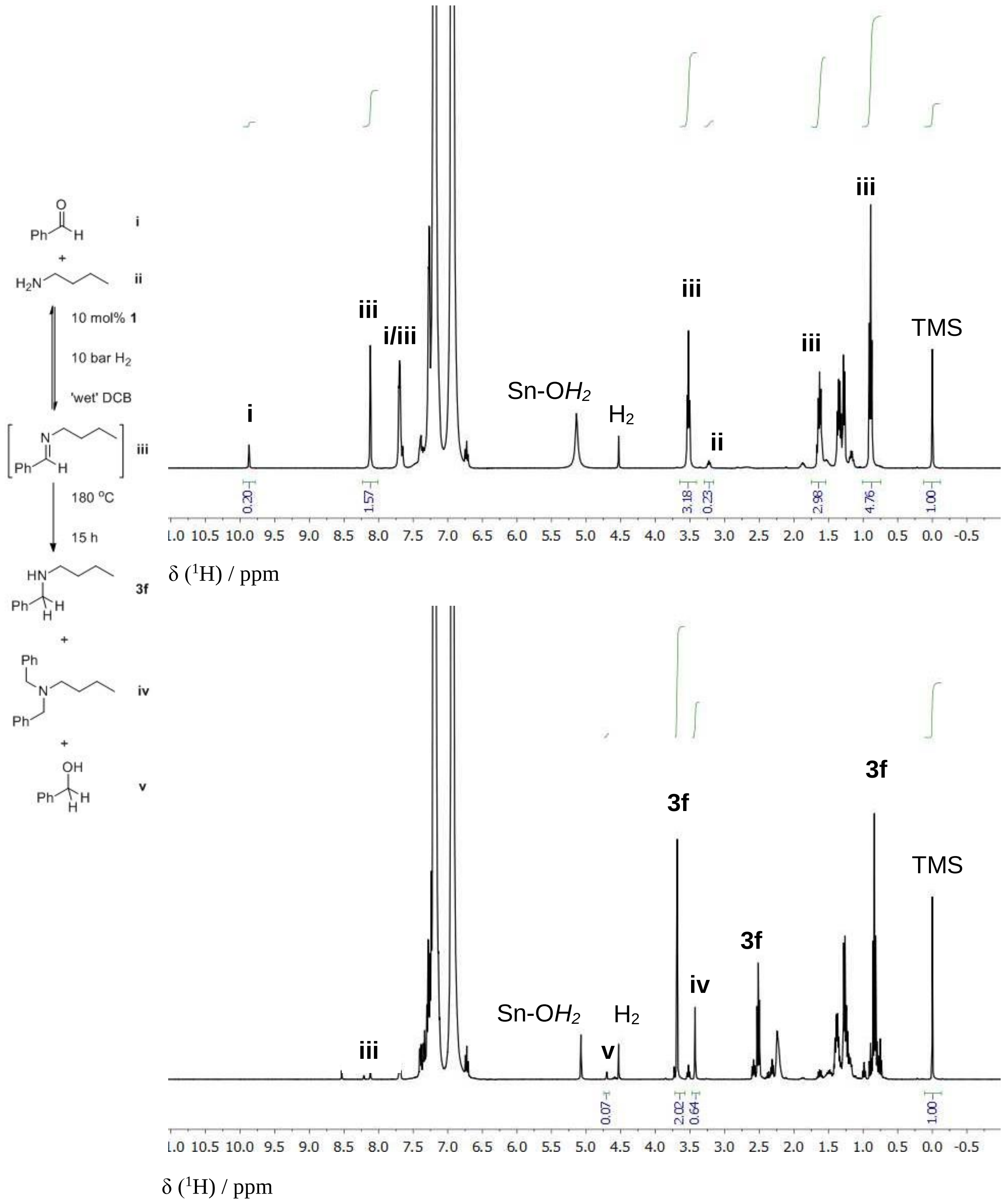

Figure S8a: ^{1}H NMR spectra for the reductive amination to **3f**. Conversion (%): **3f** (59), **iv** (9), **v** (2). Presence of products confirmed by comparison to spectral data of pure, authentic compounds where possible: **iii**,[13] **3f**,[3] **iv**,[14] **v**.[2] **iv** arises from over-alkylation (RA of **3f** and **i**).

3f ^{1}H NMR (400 MHz): δ = 3.69 (s, 2H, N–CH_2–Ph), 2.51 (t, $^{3}J_{HH}$ = 7.1 Hz, 2H, N–CH_2–CH_2), 1.42-1.22 (m, 4H, N–CH_2–CH_2–CH_2), 0.84 (t, $^{3}J_{HH}$ = 7.3 Hz, 3H, N–$(CH_2)_3$–CH_3).

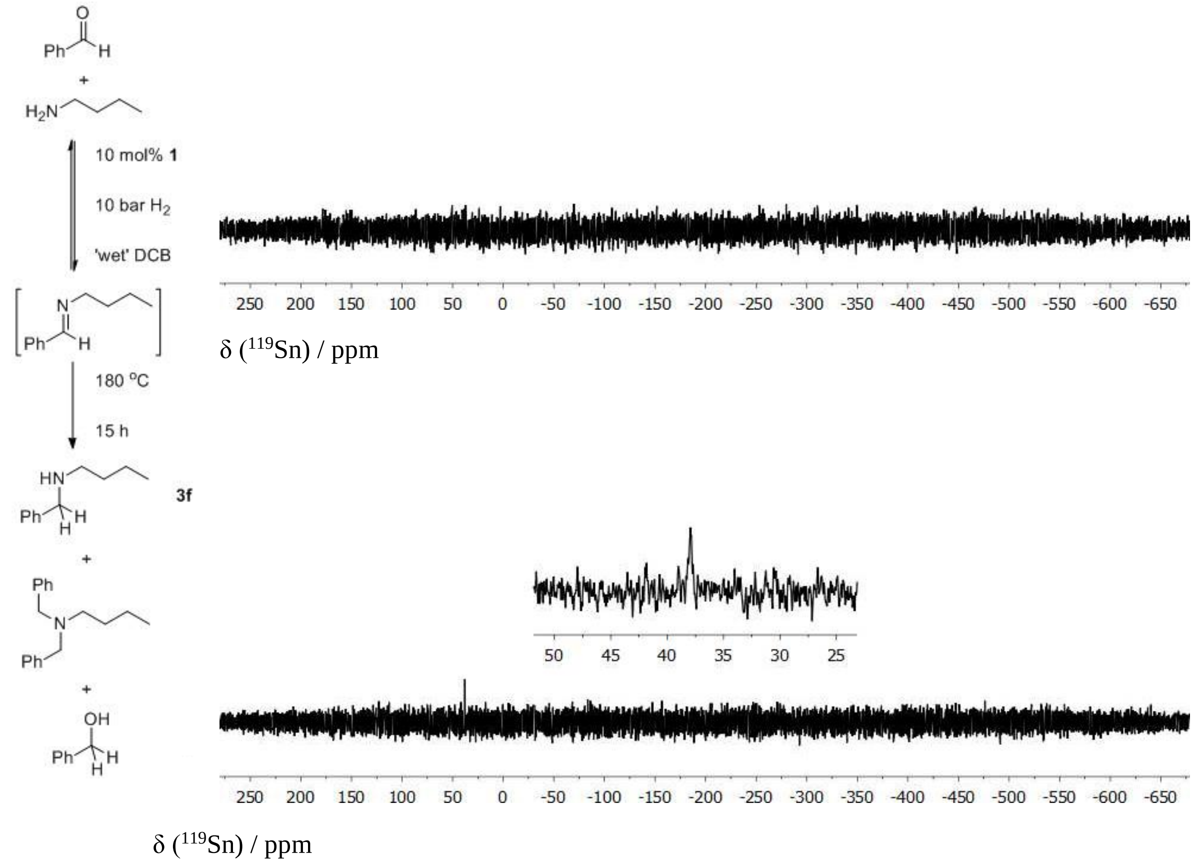

Figure S8b: $^{119}Sn\{^{1}H\}$ NMR spectra for the reductive amination to **3f**. Inset provides expanded view of the observed resonance.

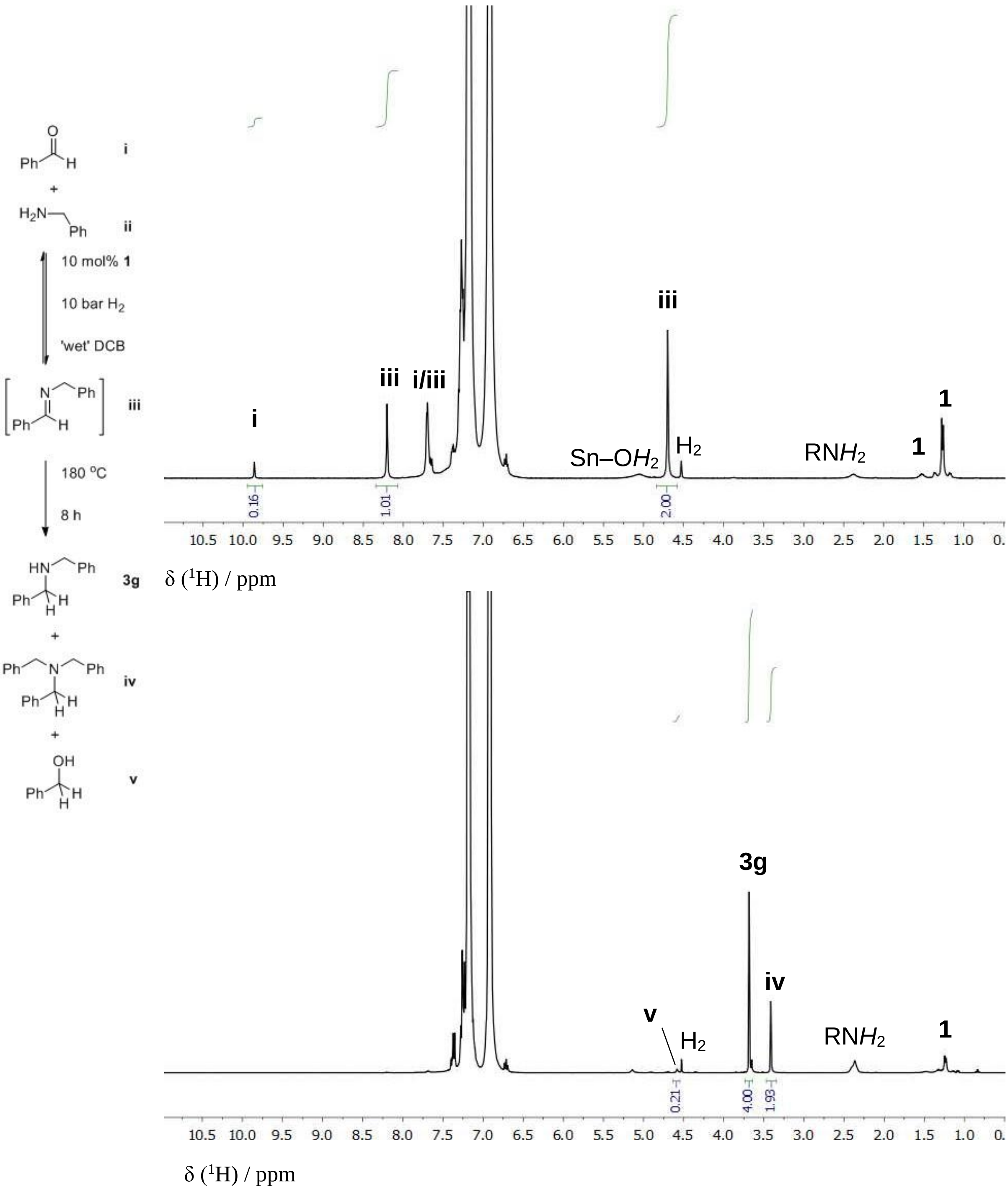

Figure S9: ^{1}H NMR spectra for the reductive amination to **3g**. Conversion (%): **3g** (70), **iv** (23), **v** (7). Presence of products confirmed by comparison to spectral data of pure, authentic compounds: **iii**,[15] **3g**,[2] **iv**,[16] **v**.[2] **iv** arises from over-alkylation (RA of **3g** and **i**).

3g ^{1}H NMR (400 MHz): δ = 3.68 (s, 4H, N–(CH_2–Ph)$_2$), 2.37 (br s, 1H, NH).

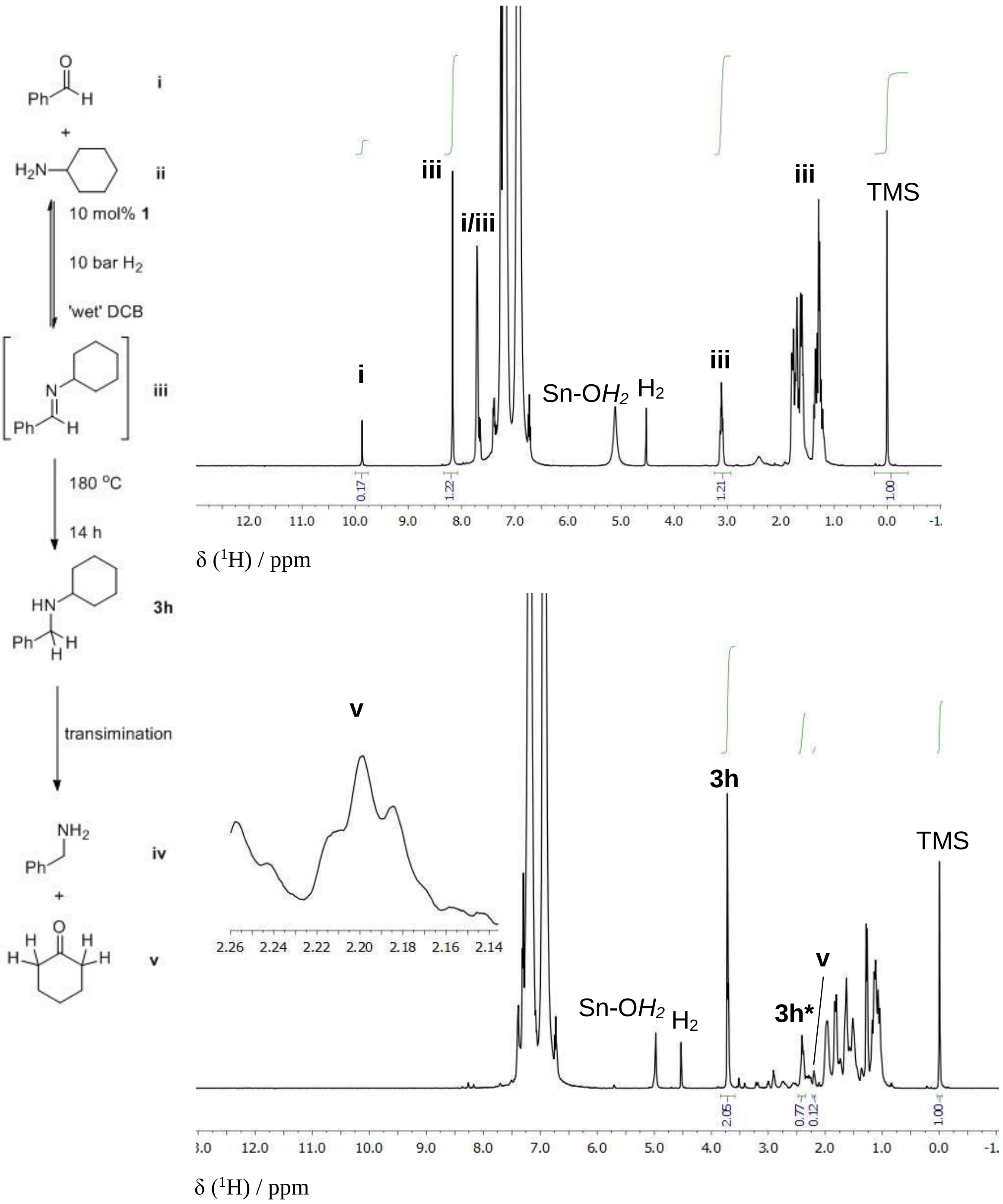

Figure S10a: ^{1}H NMR spectra for the reductive amination to **3h**. Conversion (%): **3h** (75). Presence of products confirmed by comparison to spectral data of pure, authentic compounds : **iii**,[17] **3h**.[18] Presence of **v** confirmed by comparison to authentic sample in DCB. Transimination of **3h** results in **iv** and **v**, although **iv** is not observed. Presumably **iv** attacks **i** to form a variety of products (RA of **iv** and **i**, the product of which could undergo further RA with **i**). Inset shows expanded view of the observed **v** resonance.

***** the only visible **3h** cyclohexyl resonance overlaps with cyclohexyl resonances of other species.

3h ^{1}H NMR (400 MHz): δ = 3.72 (s, 2H, N–CH_2–Ph), 2.41 (m, 1H, cyclohexyl N–C*H*–(CH_2)$_2$), 1.98-1.04 (m, 10H, cyclohexyl N–CH–(CH_2)$_5$).

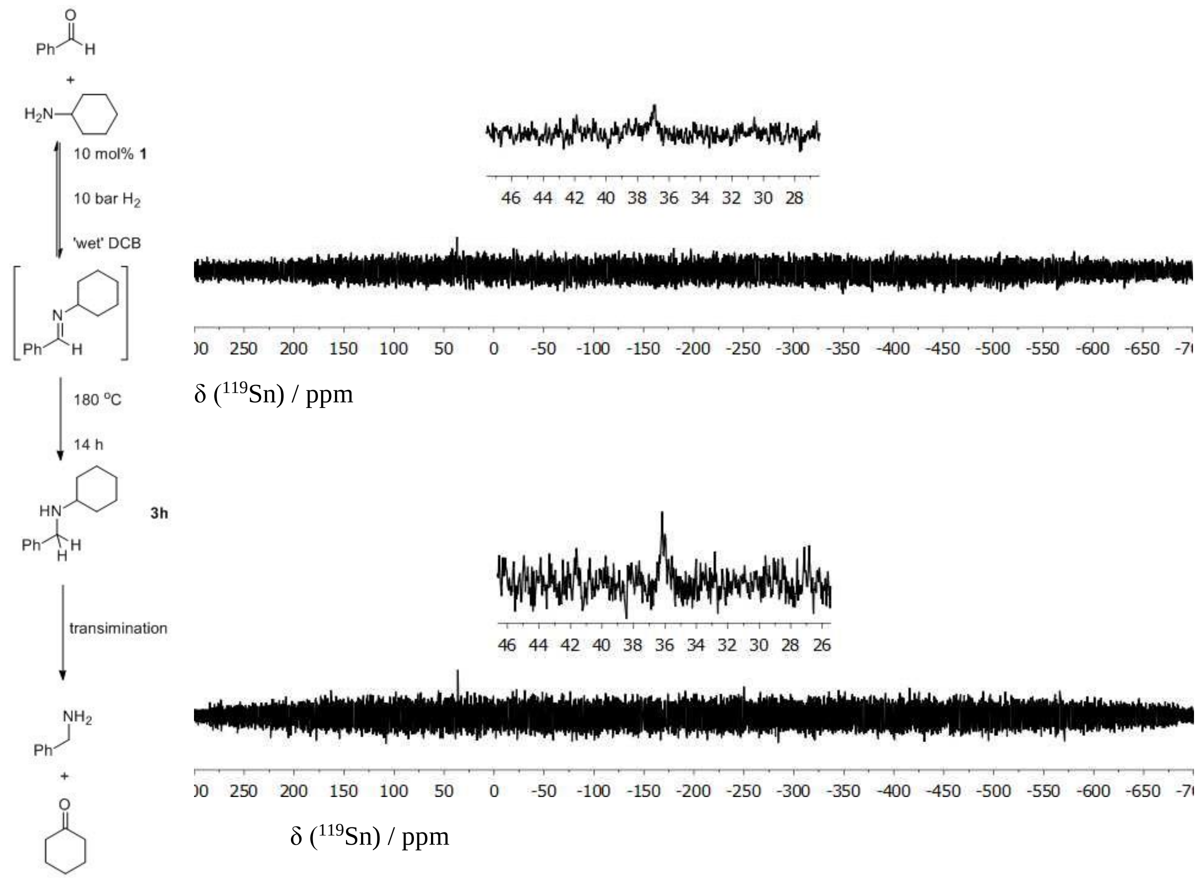

Figure S10b: $^{119}Sn\{^{1}H\}$ NMR spectra for the reductive amination to **3h**. Inset provides expanded view of the observed resonance.

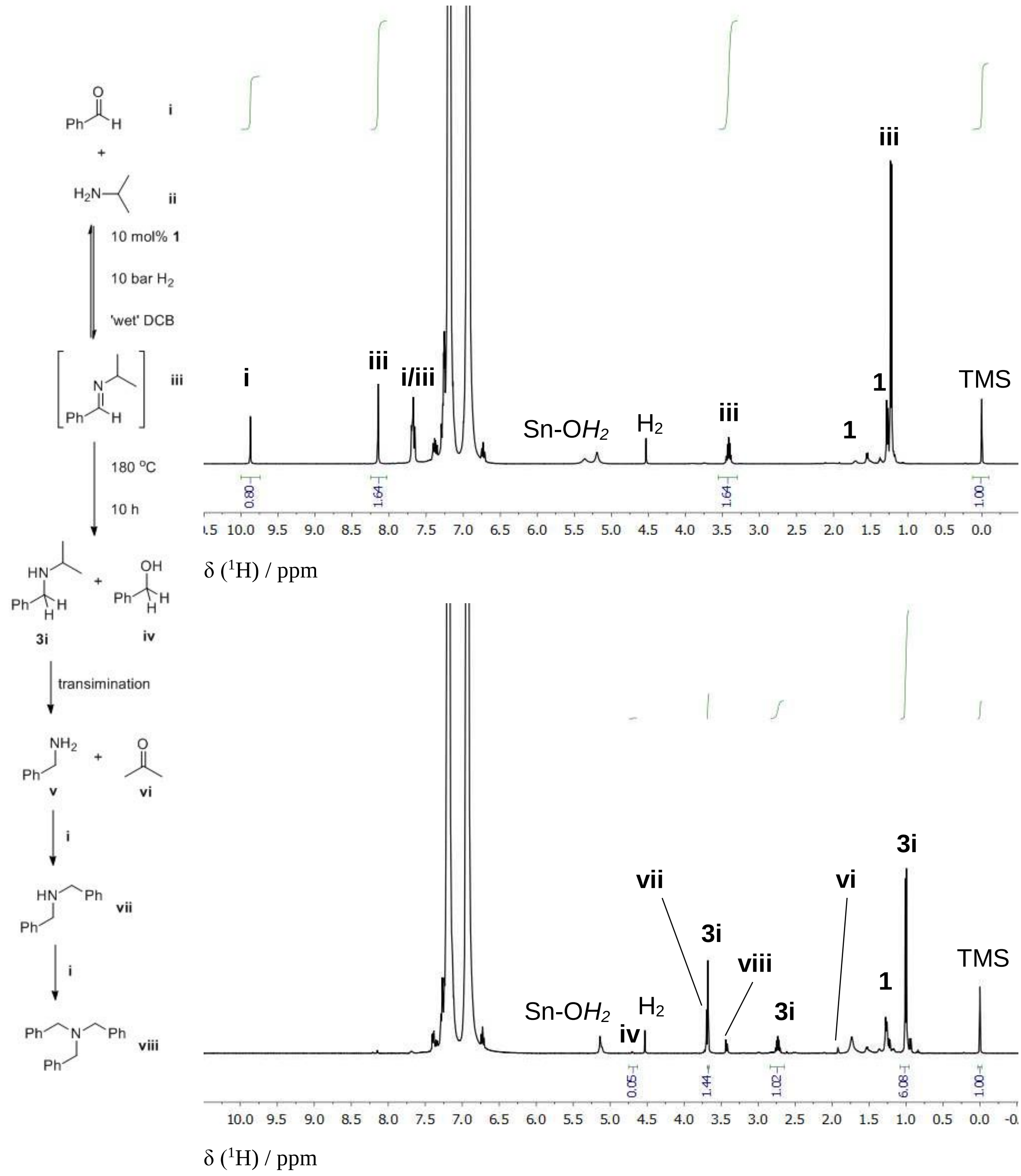

Figure S11a: ^{1}H NMR spectra for the reductive amination to **3i**. Conversion (%): **3i** (62), **iv** (2). Presence of products confirmed by comparison to spectral data of pure, authentic compounds: **iii**[5], **3i**,[19] **iv**,[2] **vii**,[2] **viii**.[16] Transimination of **3i** results in **v** and **vi**; although **iv** is not directly observed. **v** attacks **i** to form a variety of products (RA of **v** and **i** forms **vii**, which undergoes further RA with **i** to form **viii**).

3i ^{1}H NMR (400 MHz): δ = 3.68 (s, 2H, N–C*H*$_2$–Ph), 2.73 (sept, $^{3}J_{HH}$ = 6.2 Hz, 1H, N–C*H*$_2$–(CH$_3$)$_2$), 1.00 (d, $^{3}J_{HH}$ = 6.2 Hz, 6H, N–CH$_2$–(C*H*$_3$)$_2$).

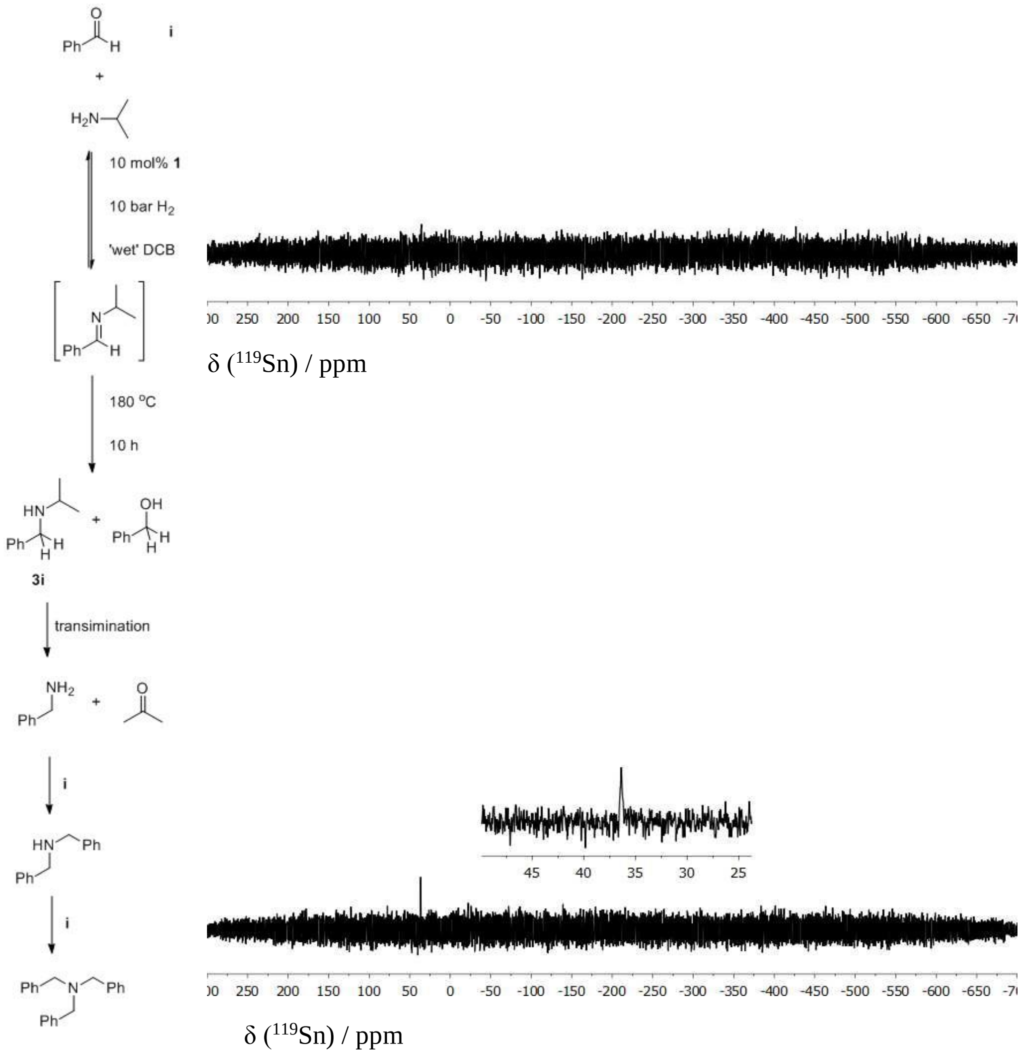

Figure S11b: $^{119}Sn\{^{1}H\}$ NMR spectra for the reductive amination to **3i**. Inset provides expanded view of the observed broad resonance.

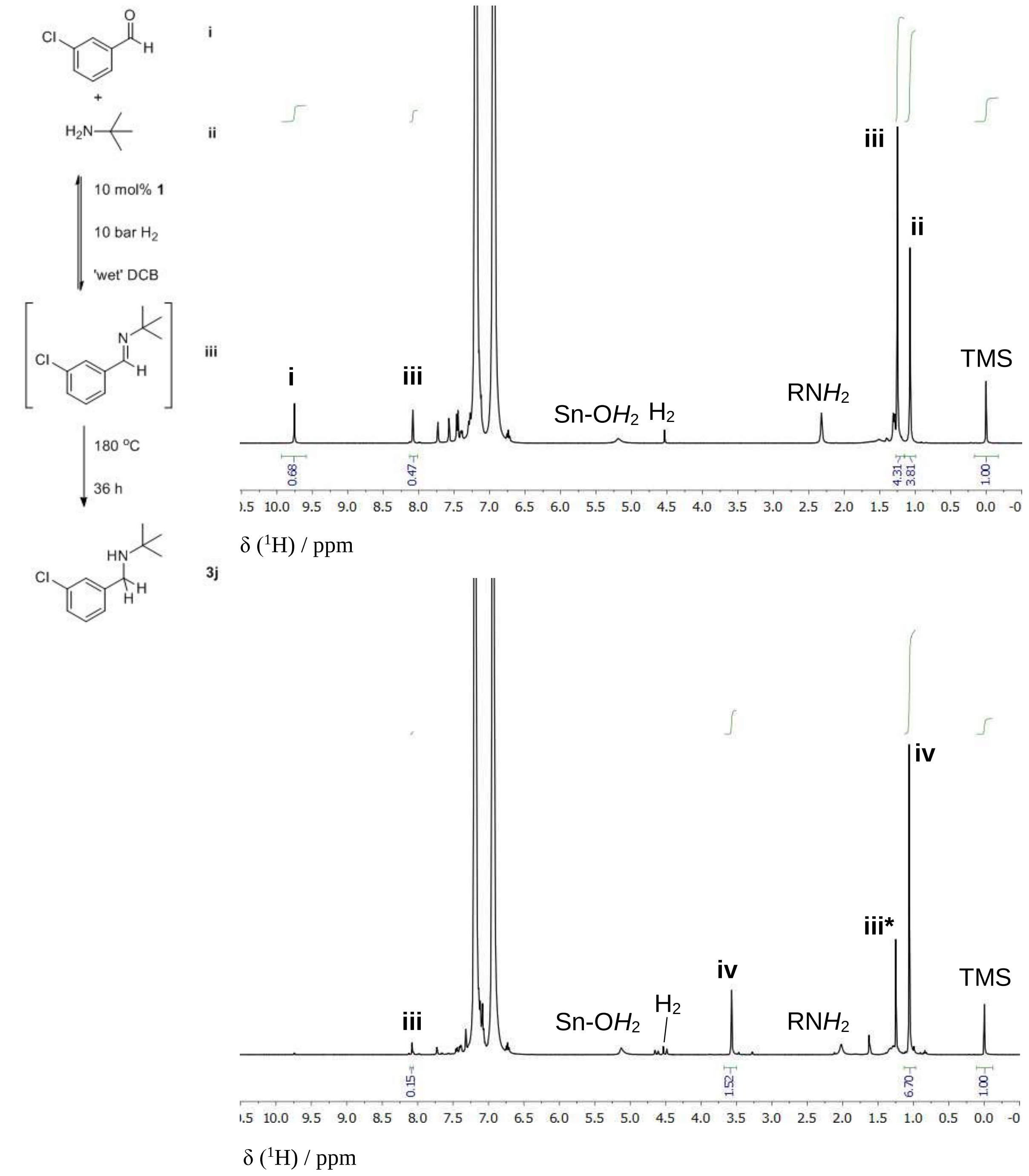

Figure S12: ^{1}H NMR spectra for the reductive amination to **3j**. Conversion (%): **3j** (85). Presence of products confirmed by comparison to spectral data of pure, authentic compounds: **iii**,[5] **3j**.[20]

iii* peak overlaps with **1** SnCH(CH_3)$_2$ peaks.

3j ^{1}H NMR (400 MHz): δ = 3.57 (s, 2H, N–CH_2–Ph), 2.02 (s, 1H, NH), 1.06 (s, 9H, N–C(CH_3)$_3$).

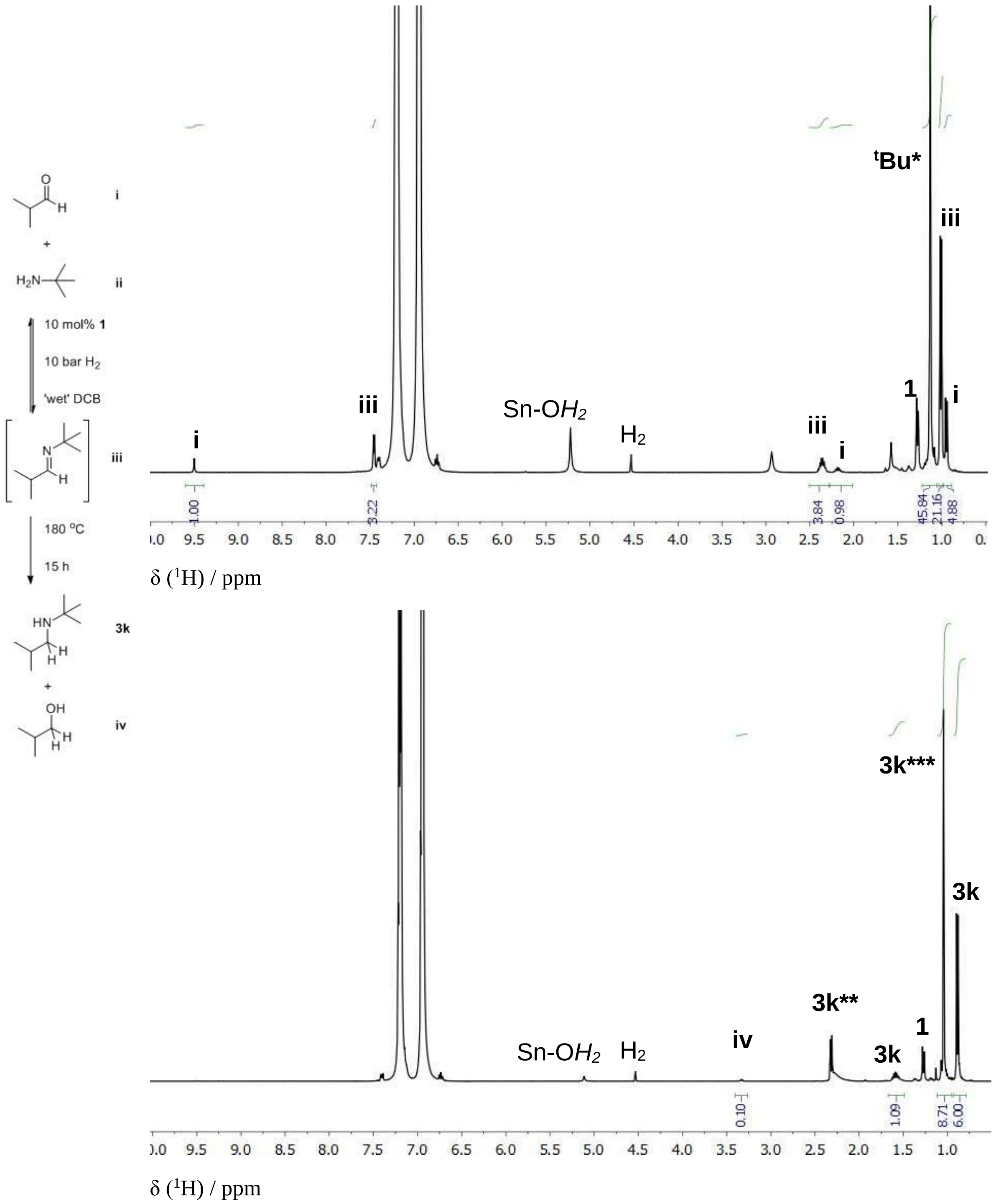

Figure S13a: ^{1}H NMR spectra for the reductive amination to **3k**. Conversion (%): **3k** (95), **iv** (5). Presence of products confirmed by comparison to spectral data of pure, authentic compounds where possible: **iii**,[21] **iv**.[22]

* ^{t}**Bu** resonances for **ii** and **iii** are identical, and appear as one peak.
** **3k** CH(C*H*$_3$)$_2$ peak overlaps with RN*H* resonance.
*** **3k** ^{t}Bu resonance overlaps with residual **ii**.

3k ^{1}H NMR (400 MHz): δ = 2.32 (d, $^{3}J_{HH}$ = 6.6 Hz, 2H, N–C*H*$_2$), 2.28 (br s, 1H, N*H*), 1.64-1.54 (m, 1H, N–CH$_2$–C*H*–(CH$_3$)$_2$), 1.04 (s, 9H, N–C(C*H*$_3$)$_3$), 0.89 (d, $^{3}J_{HH}$ = 6.6 Hz, 6H, N–CH$_2$–CH–(C*H*$_3$)$_2$).

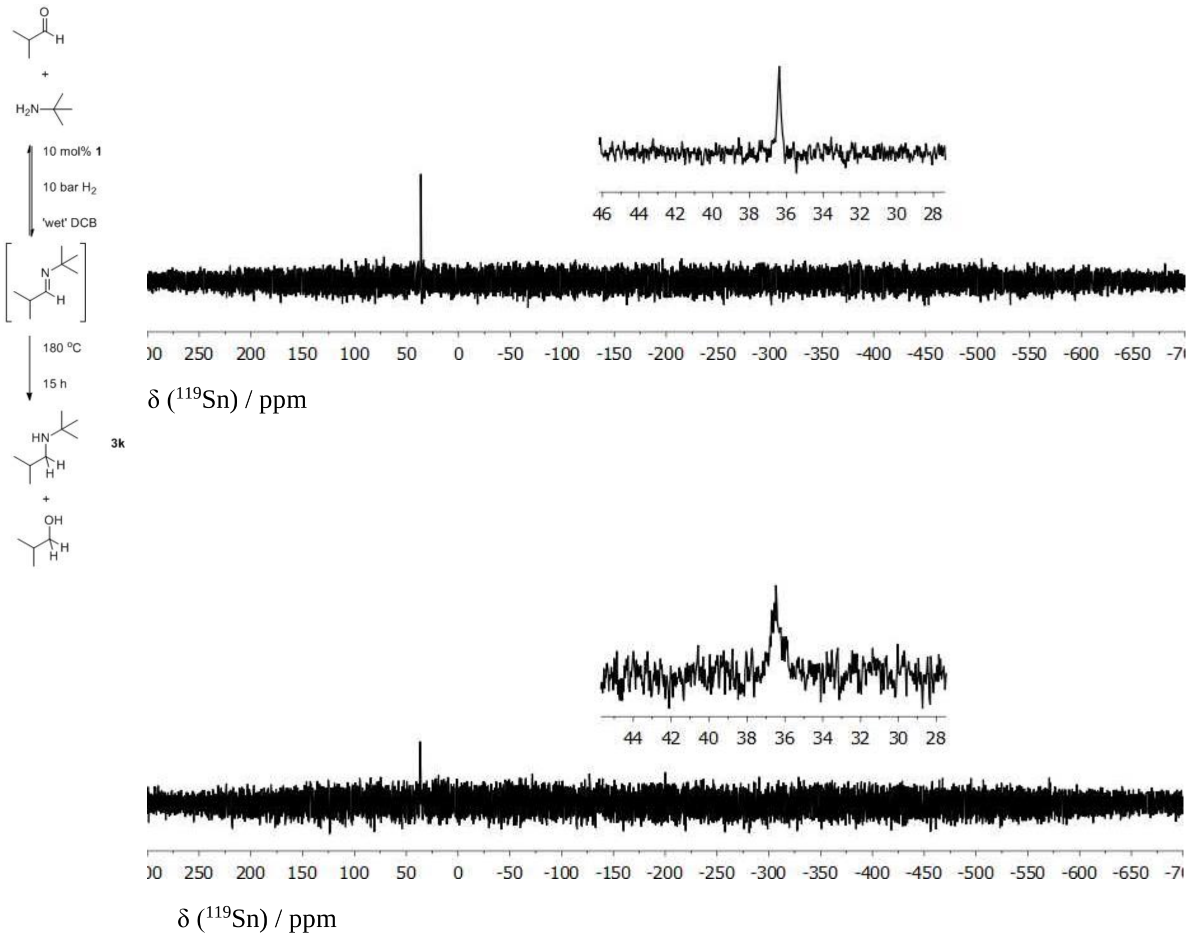

Figure S13b: ^{119}Sn {^{1}H} NMR spectra for the reductive amination to **3k**. Inset provides expanded view of the observed broad resonance.

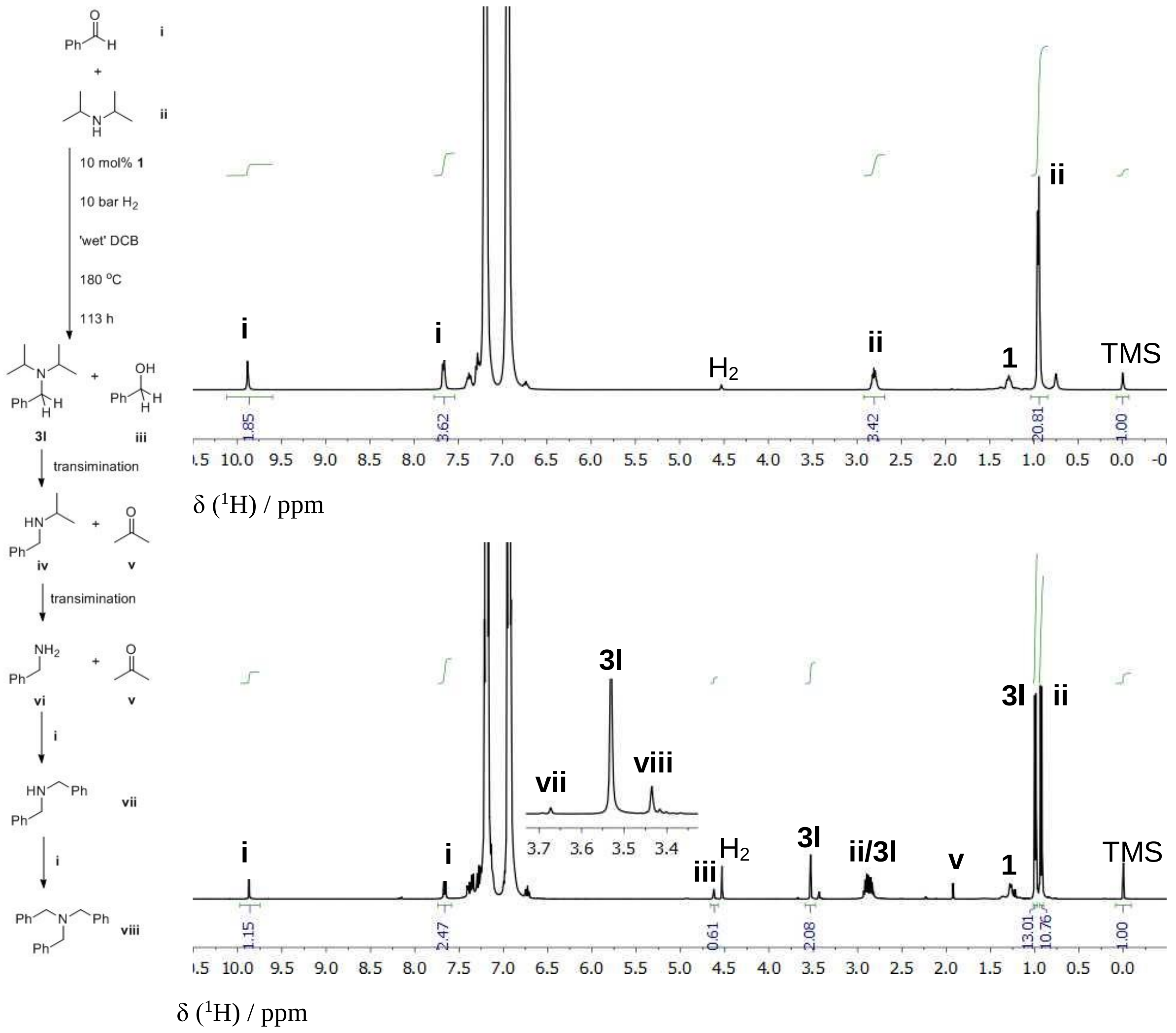

Figure S14a: ^{1}H NMR spectra for the reductive amination to **3l**. Conversion (%): **3l** (61), **iii** (18). Presence of products confirmed by comparison to spectral data of pure, authentic compounds: **3l**,[23] **iii**,[2] **vii**,[2] **viii**.[16] Transimination of **3l** results in **iv** and **v**. **iv** undergoes a second transimination to form **vi**. **vi** then attacks **i** (RA of **vi** and **i** forms **vii**, which undergoes further RA with **i** to form **viii**). Inset provides expanded view of the PhCH_2N region of the spectrum.

3l ^{1}H NMR (400 MHz): δ = 3.53 (s, 2H, N–CH_2–Ph), 2.93-2.82 (m, 2H, N–(CH–(CH$_3$)$_2$)$_2$), 1.00 (d, $^{3}J_{HH}$ = 6.3 Hz, 12H, N–(CH–(CH_3)$_2$)$_2$).

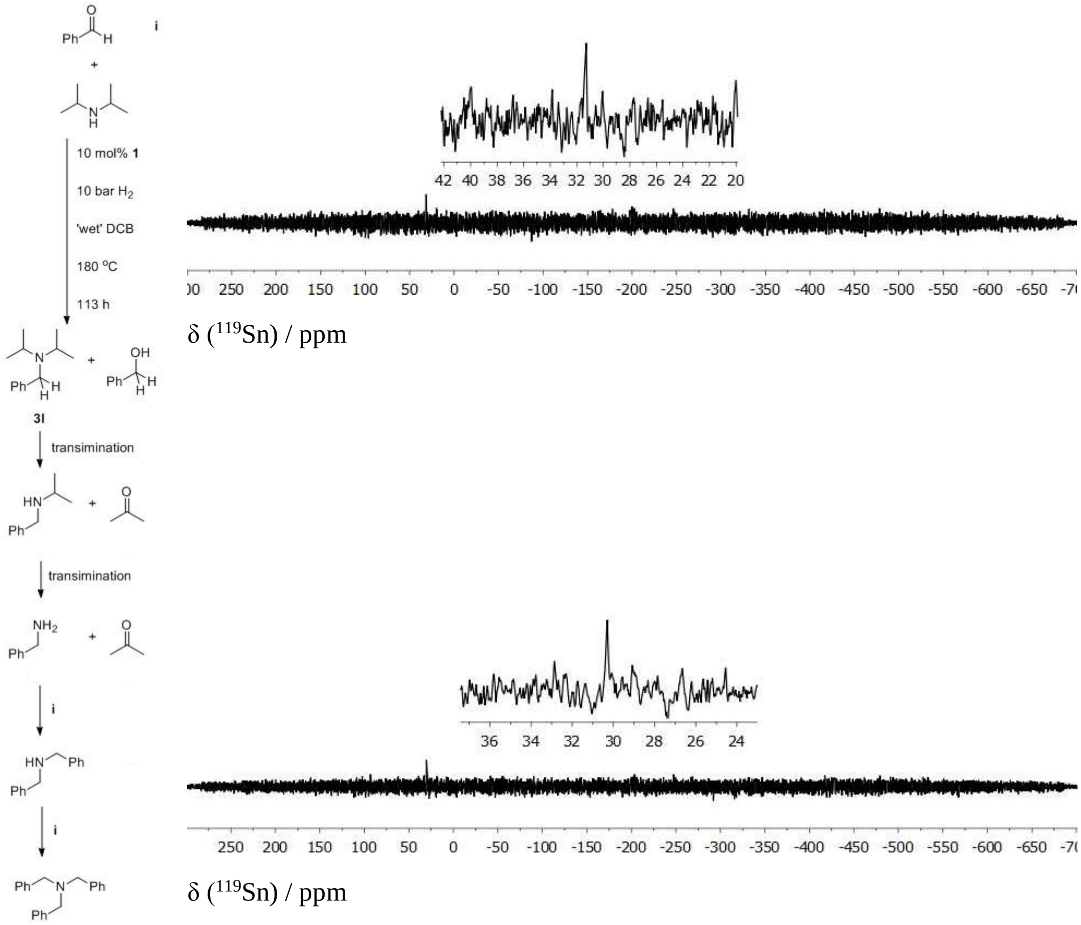

Figure S14b: $^{119}Sn\{^{1}H\}$ NMR spectra for the reductive amination to **3l**. Inset provides expanded view of the observed broad resonance.

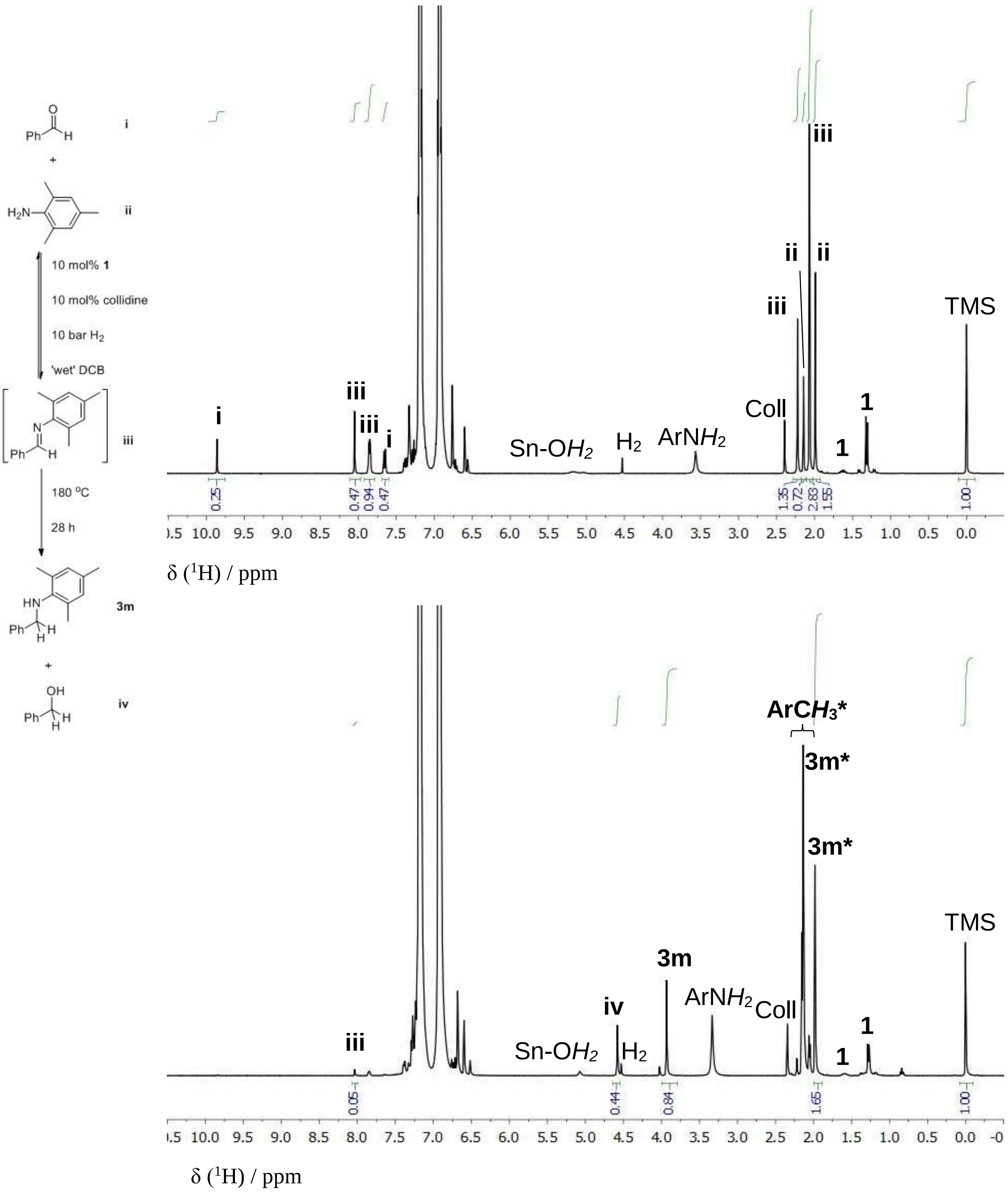

Figure S15: ^{1}H NMR spectra for the hydrogenation to **3m**. Conversion (%): **3m** (58), **iv** (31). Presence of products confirmed by comparison to spectral data of pure, authentic compounds: **iii**,[24] **3m**,[25] **iv**.[2]

* **ArC*H*₃** although the **3m*** ArC*H*₃ resonances are clearly visible, they overlap with ArC*H*₃ resonances for collidine and residual **ii**.

3m ^{1}H NMR (400 MHz): δ = 3.93 (s, 2H, N–CH_2–Ph), 2.13 (s, 6H, N–Ar–(2,6-(CH_3)$_2$)), 1.98 (s, 3H, N–Ar–(4-CH_3)).

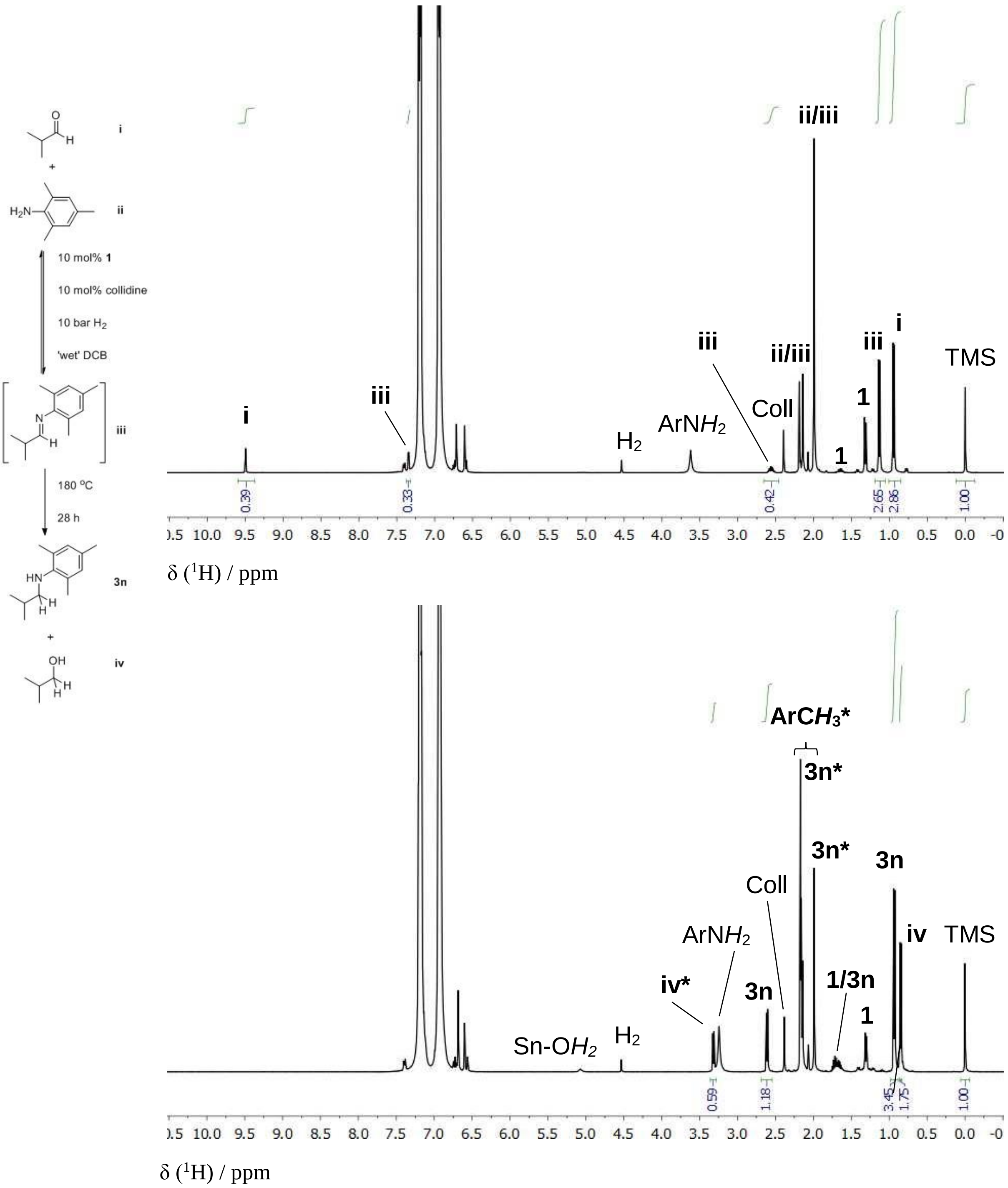

Figure S16: ^{1}H NMR spectra for the hydrogenation to **3n**. Conversion (%): **3n** (64), **iv** (32). Presence of products confirmed by comparison to spectral data of pure, authentic compounds where possible: **iii**,[26] **iv**.[27]

* **ArC*H*$_3$** although the **3n*** ArC*H*$_3$ resonances are clearly visible, they overlap with ArC*H*$_3$ resonances for collidine and residual **ii**.

3n ^{1}H NMR (400 MHz): δ = 2.61 (d, $^{3}J_{HH}$ = 6.3 Hz, 2H, N–C*H*$_2$), 2.17 (s, 6H, N–Ar–(2,6-(C*H*$_3$)$_2$)), 1.99 (s, 3H, N–Ar–(4-C*H*$_3$)), 1.75-1.64 (m, 1H, N–CH$_2$–C*H*–(CH$_3$)$_2$), 0.93 (d, $^{3}J_{HH}$ = 6.6 Hz, 6H, N–CH$_2$–CH–(C*H*$_3$)$_2$).

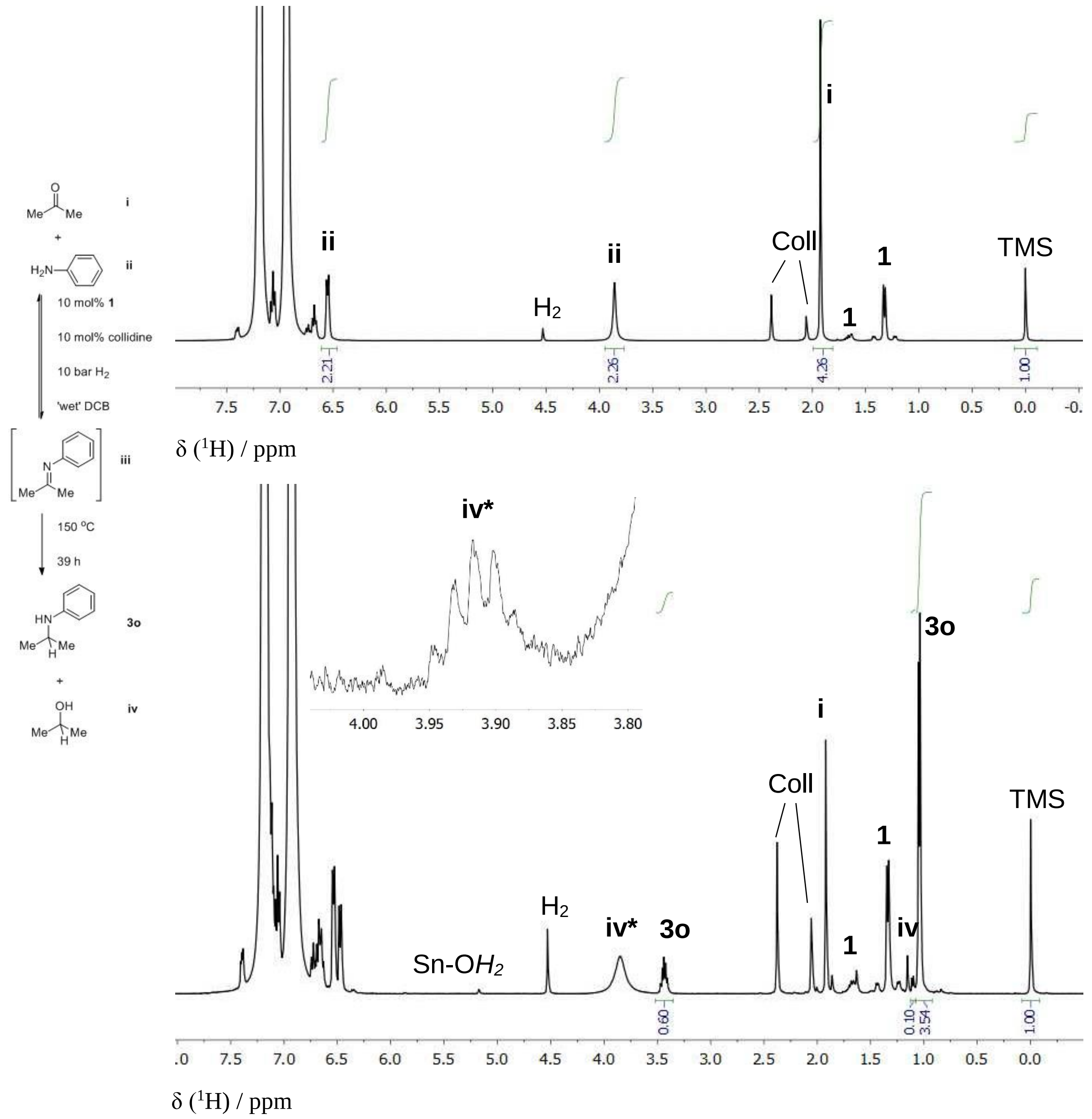

Figure S17: ^{1}H NMR spectra for the reductive amination to **3o**. Conversion (%): **3o** (85), **iv** (2). Presence of products confirmed by comparison to spectral data of pure, authentic compounds: **iii**,[28] **3o**,[29] **iv**.[30]

* **iv** HOC*H*(CH_3)$_2$ peak obscured by broad N*H* resonance; this was observed at other time points. Inset shows this peak visible at t = 16 h.

3o ^{1}H NMR (400 MHz): δ = 6.47 (d, $^{3}J_{HH}$ = 8.0 Hz, 2H, Ar–*H*) 3.44 (sept, $^{3}J_{HH}$ = 6.2 Hz, 1H, N–C*H*–(CH_3)$_2$), 1.05 (d, $^{3}J_{HH}$ = 6.2 Hz, 6H, N–CH–(C*H*$_3$)$_2$).

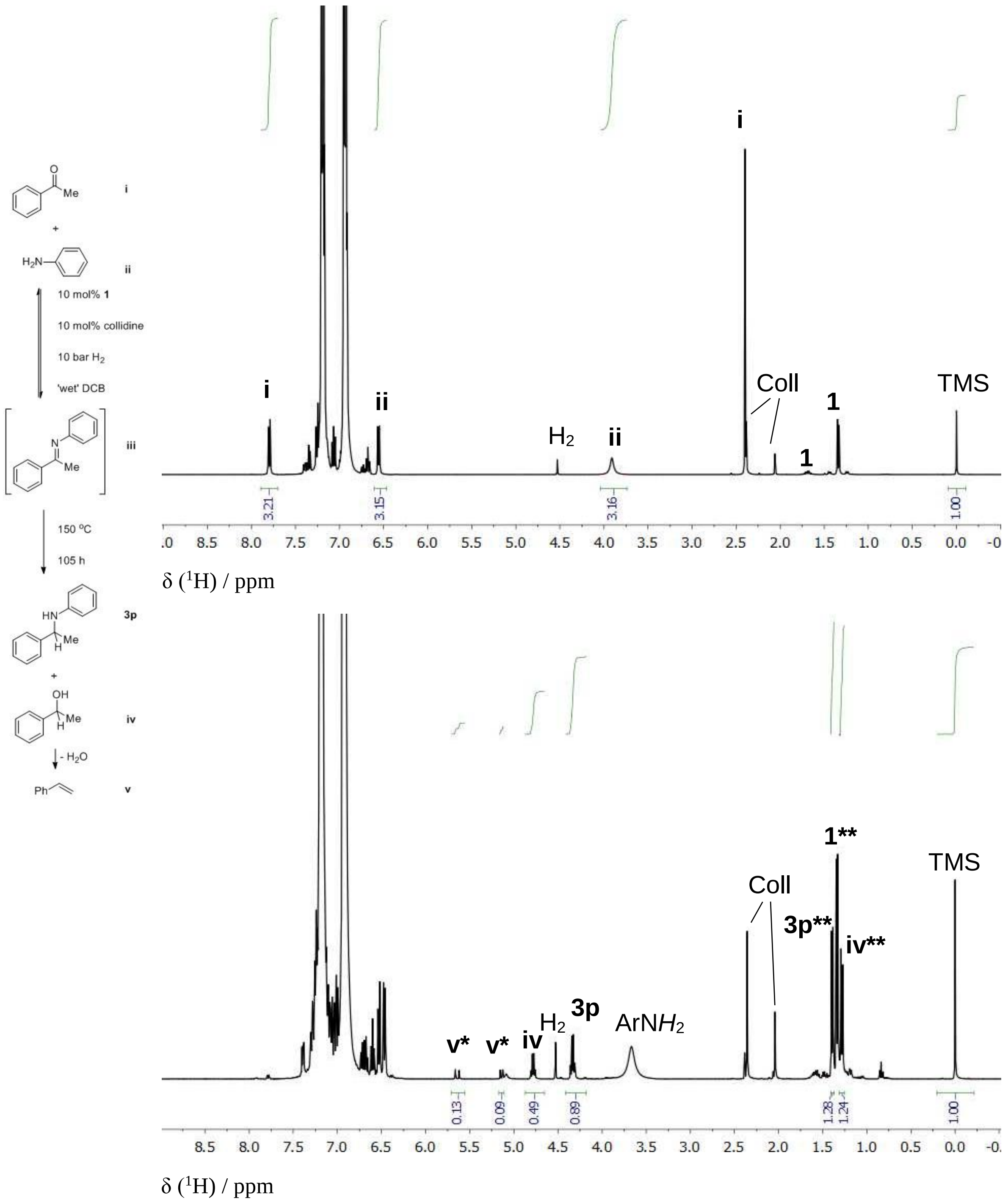

Figure S18: ^{1}H NMR spectra for the reductive amination of **3p**. Conversion (%): **3p** (56), **iv** (31), **v** (8). Presence of products confirmed by comparison to spectral data of pure, authentic compounds where possible: **iii**,[31] **3p**,[25] **iv**,[32] **v**.[33]

* **v** is generated from the dehydration of **iv** (observed during experiments concerning the direct hydrogenation of **i** using **1**).[1]

** Methyl resonances for **3p**, **1**, and **iv** overlap.

3p ^{1}H NMR (400 MHz): δ = 6.47 (d, ${}^{3}J_{HH}$ = 8.0 Hz, 2H, Ar–*H*), 4.34 (q, ${}^{3}J_{HH}$ = 6.7 Hz, 1H, N–C*H*–CH$_3$), 1.39 (d, ${}^{3}J_{HH}$ = 6.6 Hz, 3H, N–CH–C*H*$_3$).

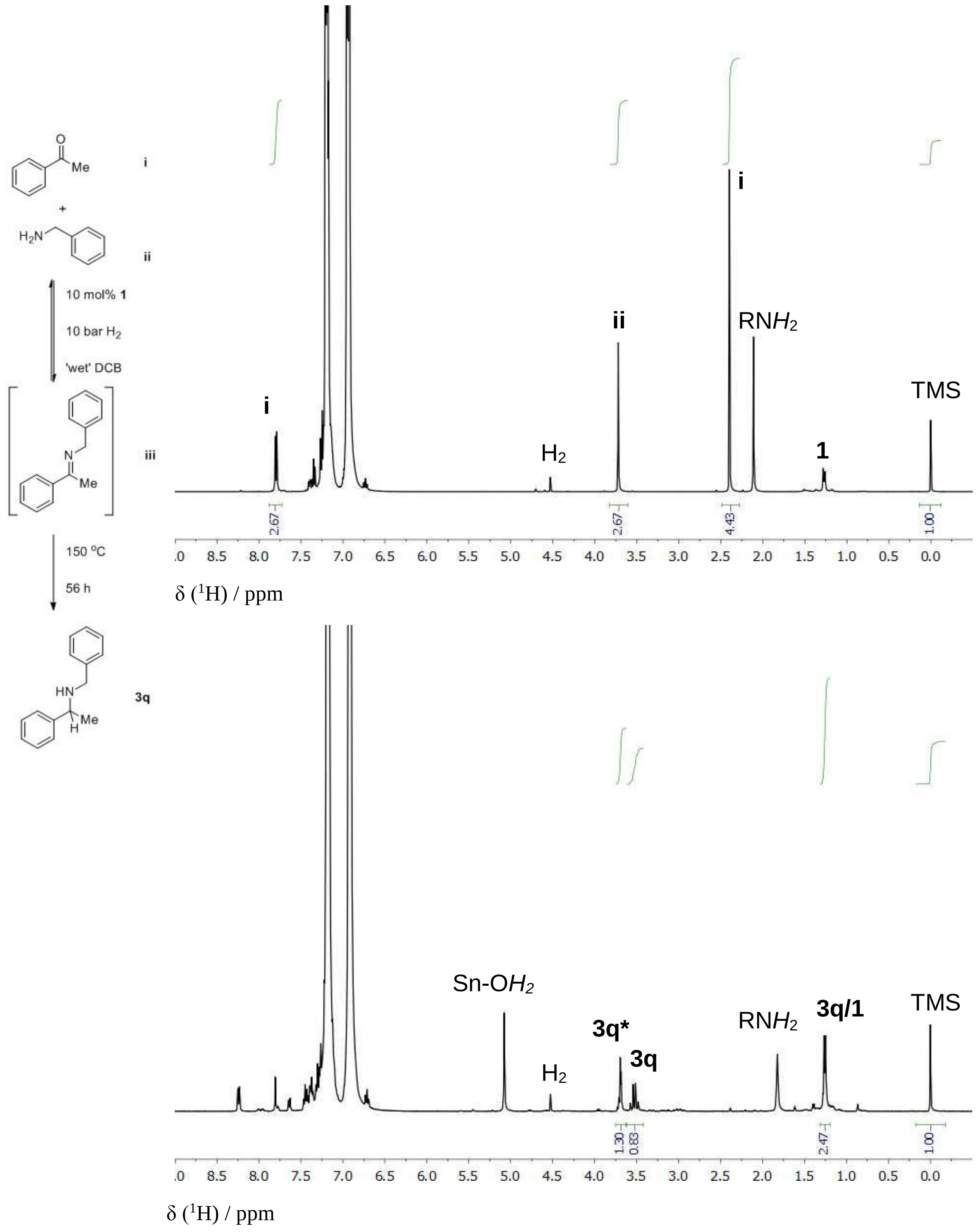

Figure S19: ^{1}H NMR spectra for the reductive amination of **3q**. Conversion (%): **3q** (62). Presence of products confirmed by comparison to spectral data of pure, authentic compounds where possible: **3q**.[34]

*** 3q** PhCH_2N resonance overlaps with residual **ii**.

3q ^{1}H NMR (400 MHz): δ = 3.71-3.69 (m, 2H, N–C*H*–Ph), 3.53 (q, $^3J_{HH}$ = 6.8 Hz, 1H, N–C*H*–CH$_3$), 1.26 (d, $^3J_{HH}$ = 6.4 Hz, 6H, N–CH–CH_3).

4. Mechanism for transimination

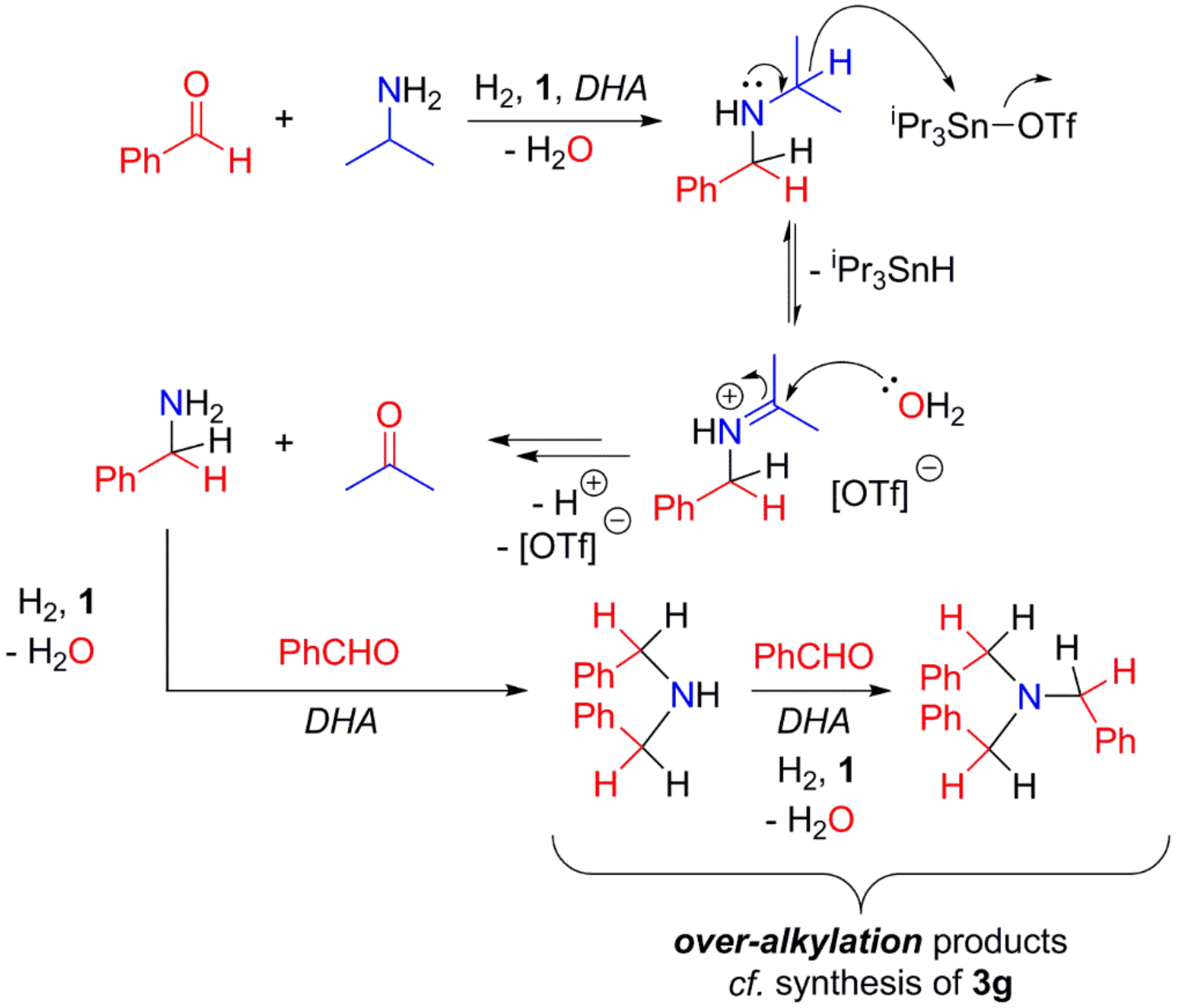

Figure S20: Transimination was observed for compounds **3h**, **3i** and **3l** (see figures S10a, S11a and S14a respectively). Note that amines formed by transimination often undergo RA with the reagent carbonyl, resulting in multiple products. Specific products are given with the NMR spectra for every reaction where transimination occurs.

5. Procedure to probe the influence of water and bases on the ^{1}H and ^{119}Sn NMR shifts of $[^{i}Pr_3Sn{\cdot}2(\mathit{H}_2O)]^{+}$

A solution of **1** (7.9 mg, 0.02 mmol), base (0.02 mmol; collidine or isopropylamine) and H_2O (1.8 µL, 0.10 mmol) was prepared in 1,2-dichlorobenzene (0.7 mL) in an NMR tube. The ^{1}H and $^{119}Sn\{^{1}H\}$ spectra of the solution were recorded. Another 1.8 µL (0.10 mmol) of H_2O was added, and the spectra were recorded again.

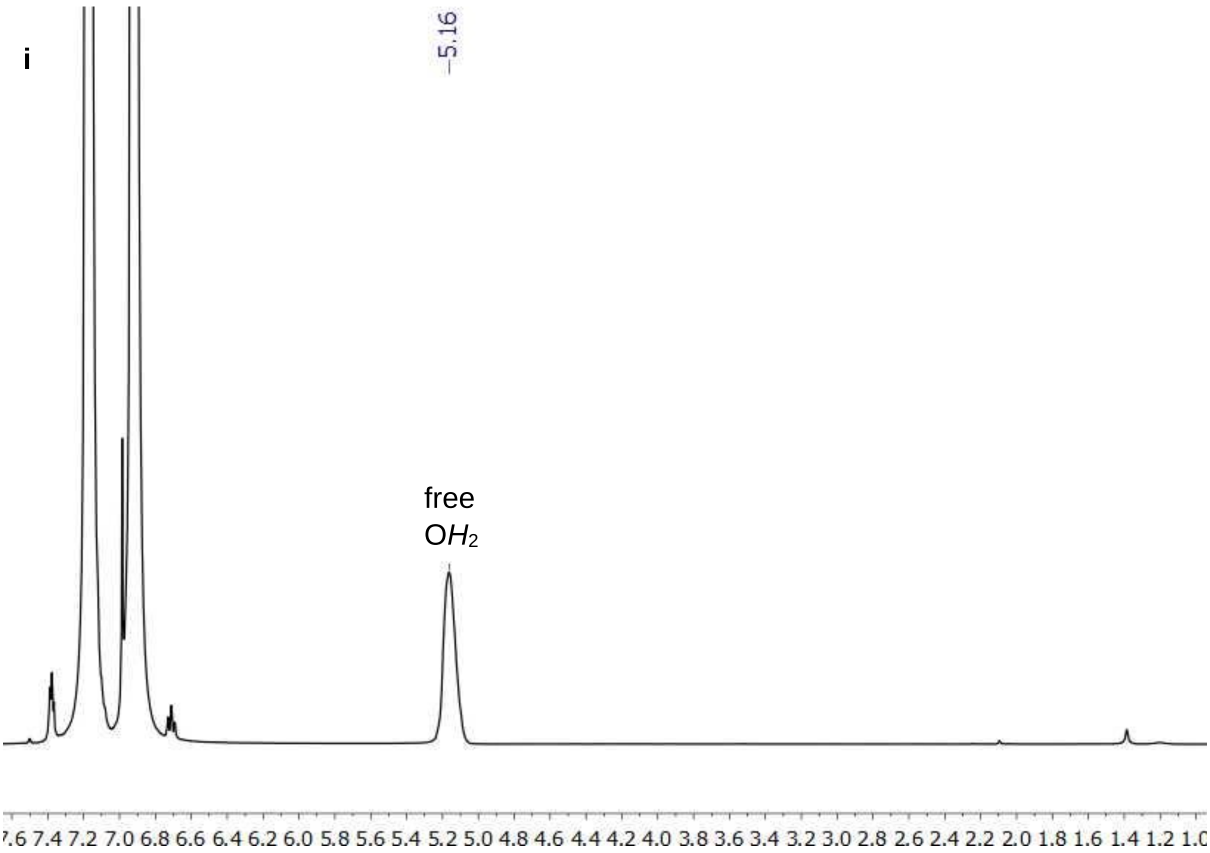

δ (^{1}H) / ppm

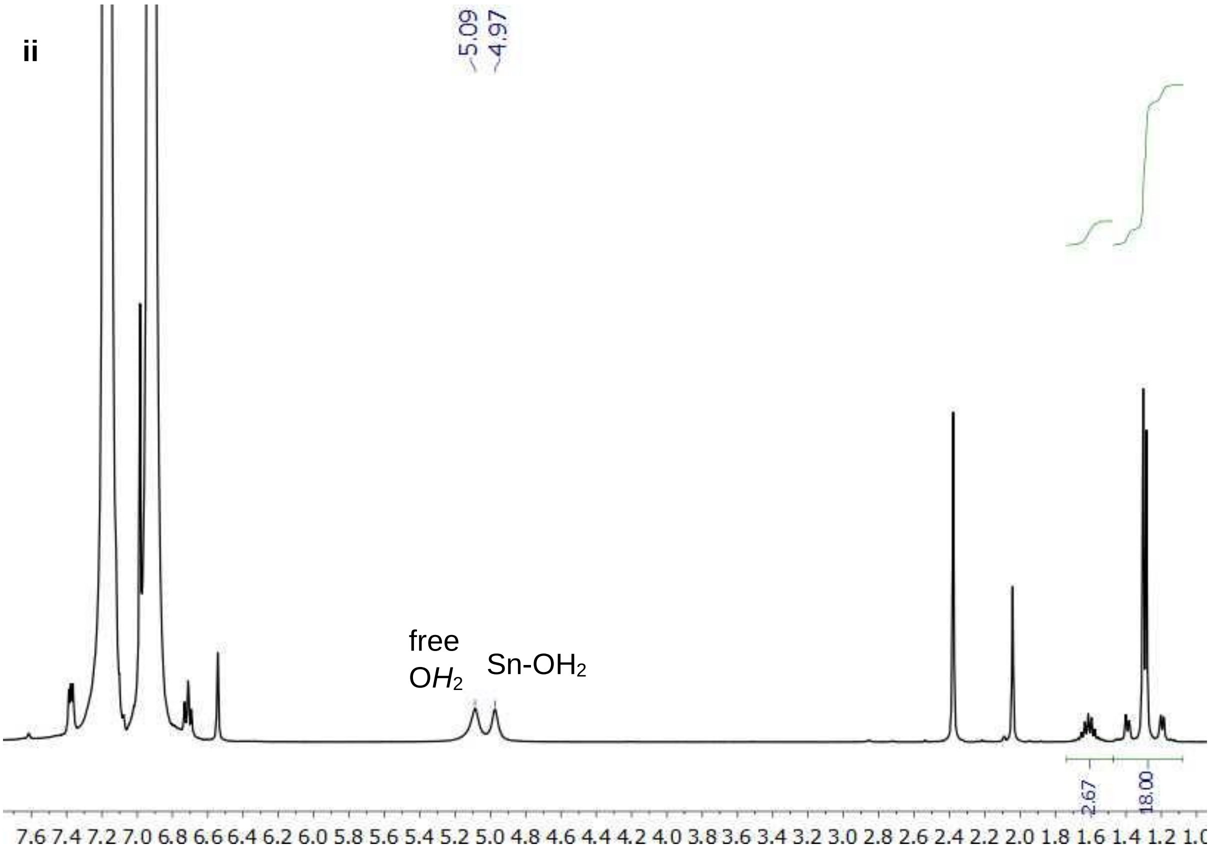

δ (^{1}H) / ppm

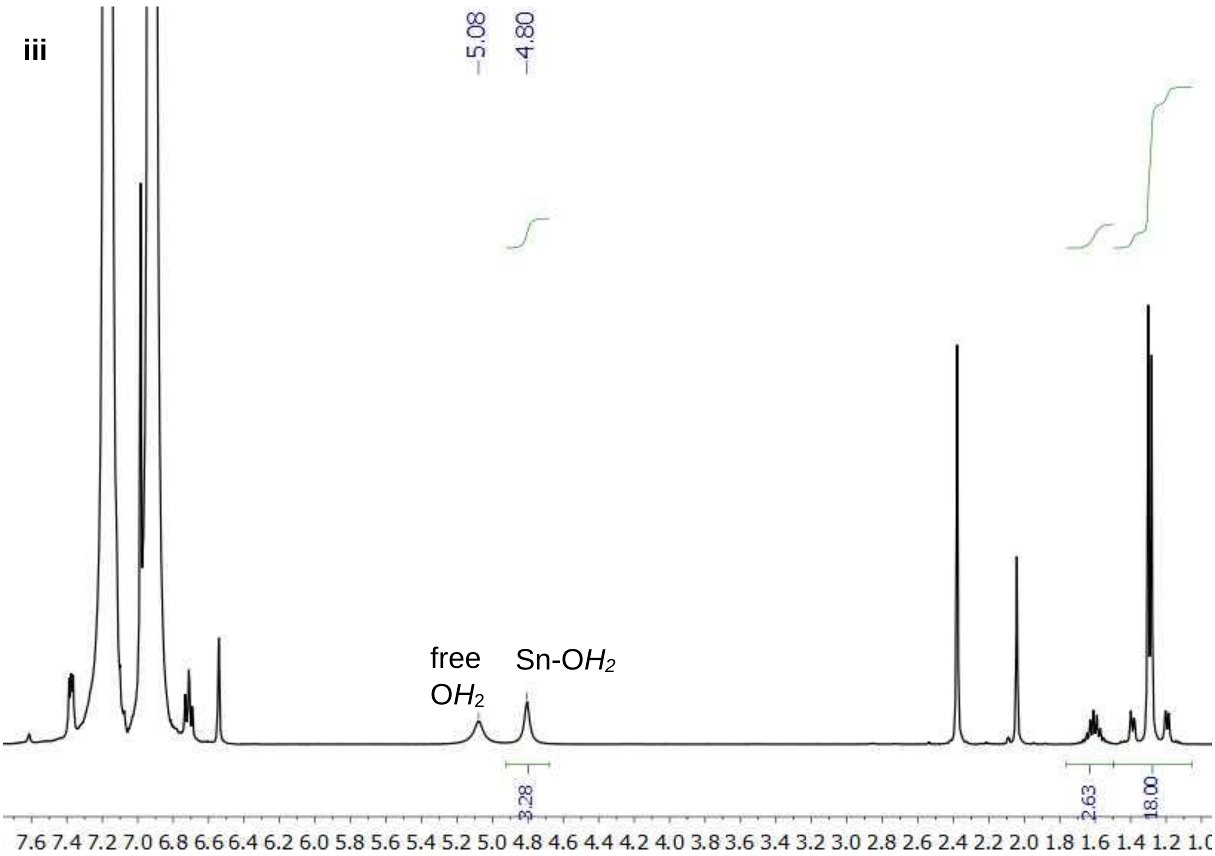

δ (^{1}H) / ppm

Figure S21a: ^{1}H NMR spectra for mixtures of **1**, collidine and H_2O in DCB.

i: H_2O in DCB

ii: **1**, collidine and H_2O (1:1:5) in DCB

iii: **1**, collidine and H_2O (1:1:10) in DCB

i

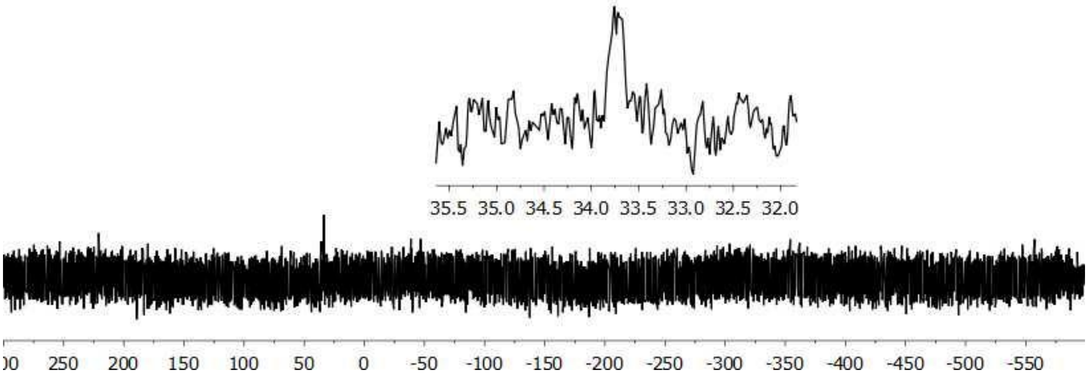

δ (^{119}Sn) / ppm

ii

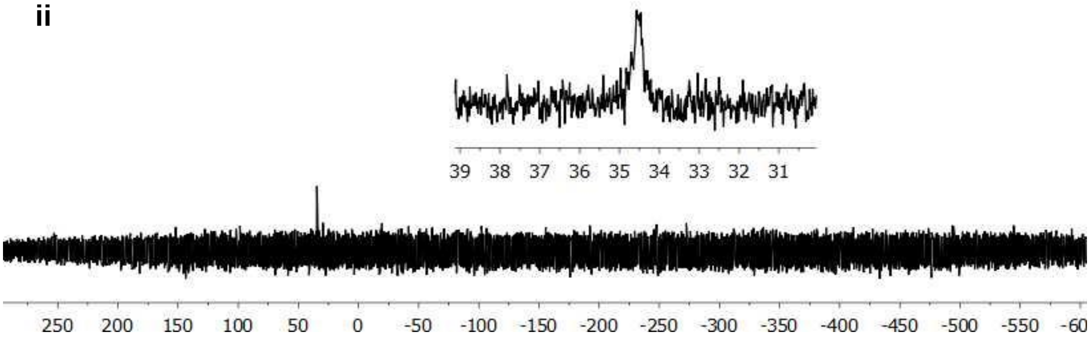

δ (^{119}Sn) / ppm

Figure S21b: ^{119}Sn{^{1}H} NMR spectra for mixtures of **1**, collidine and H_2O in DCB. **i** is of a ratio of (1:1:5); **ii** is of (1:1:10).

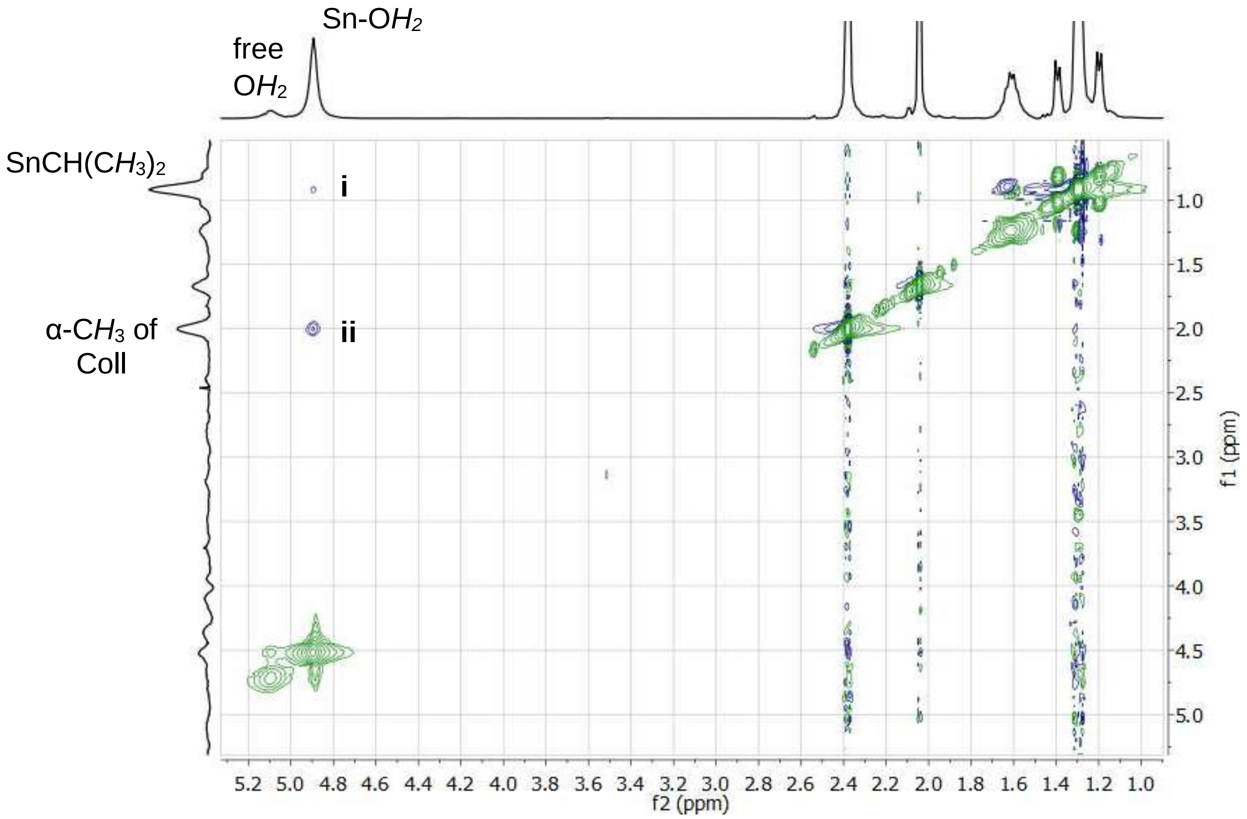

Figure S21c: NOESY spectrum of a mixture of **1**, collidine and H_2O (1:1:10) in DCB. **i** and **ii** show through-space coupling interactions:

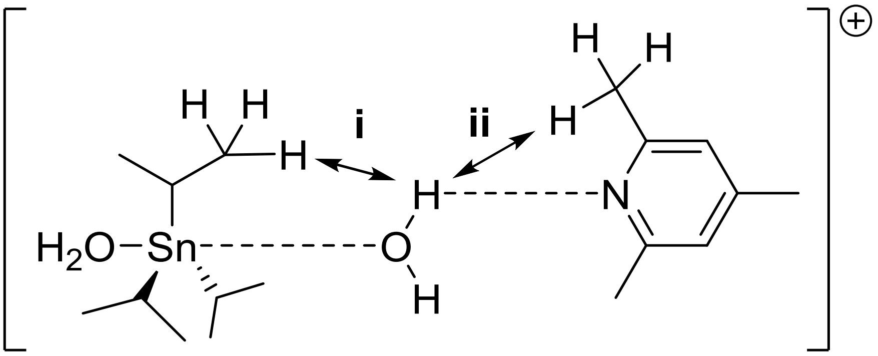

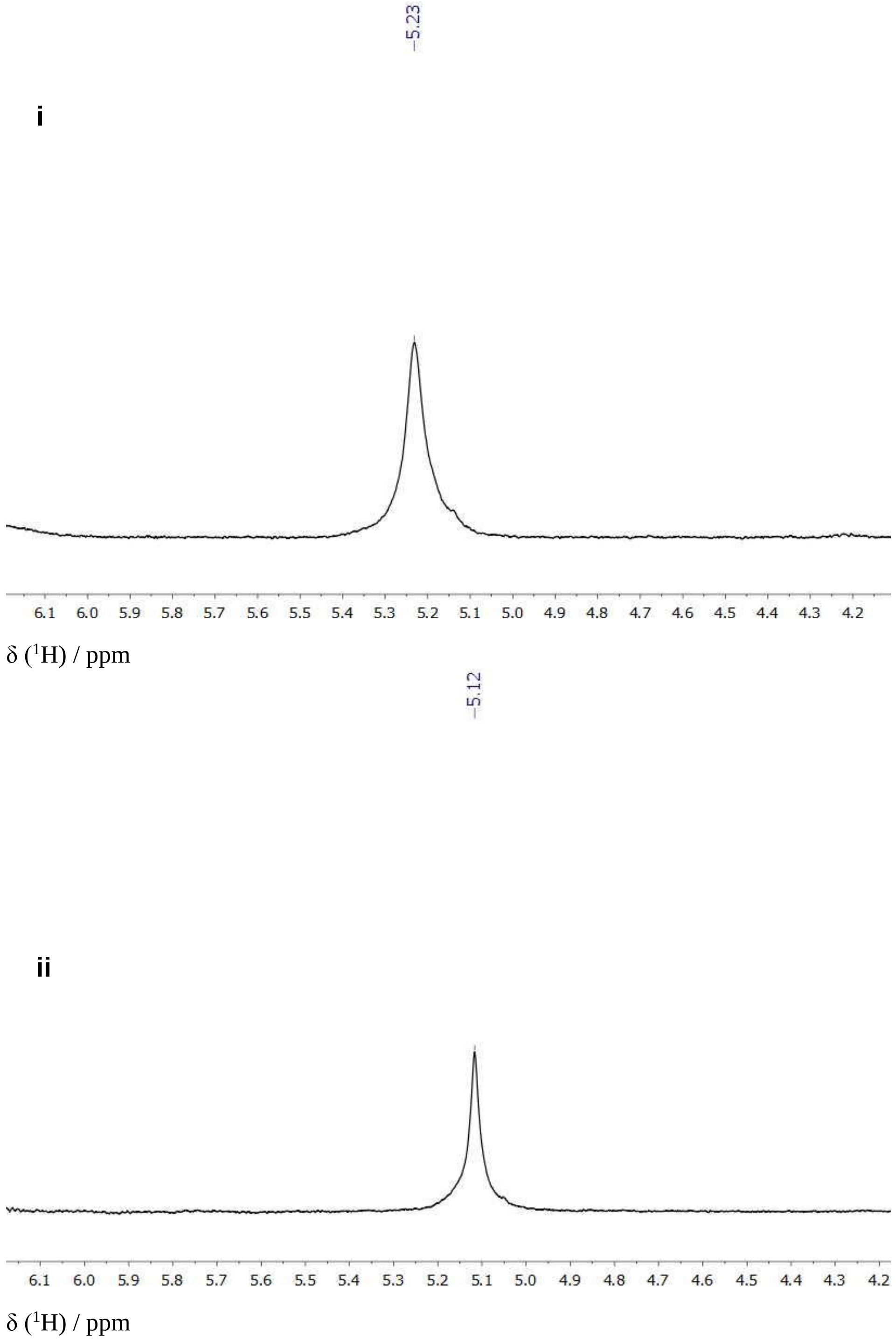

Figure S22a: ^{1}H NMR spectra of H_2O region of interest for mixtures of **1**, isopropylamine and H_2O in DCB. **i** is of a ratio of (1:1:5); **ii** is for (1:1:10).

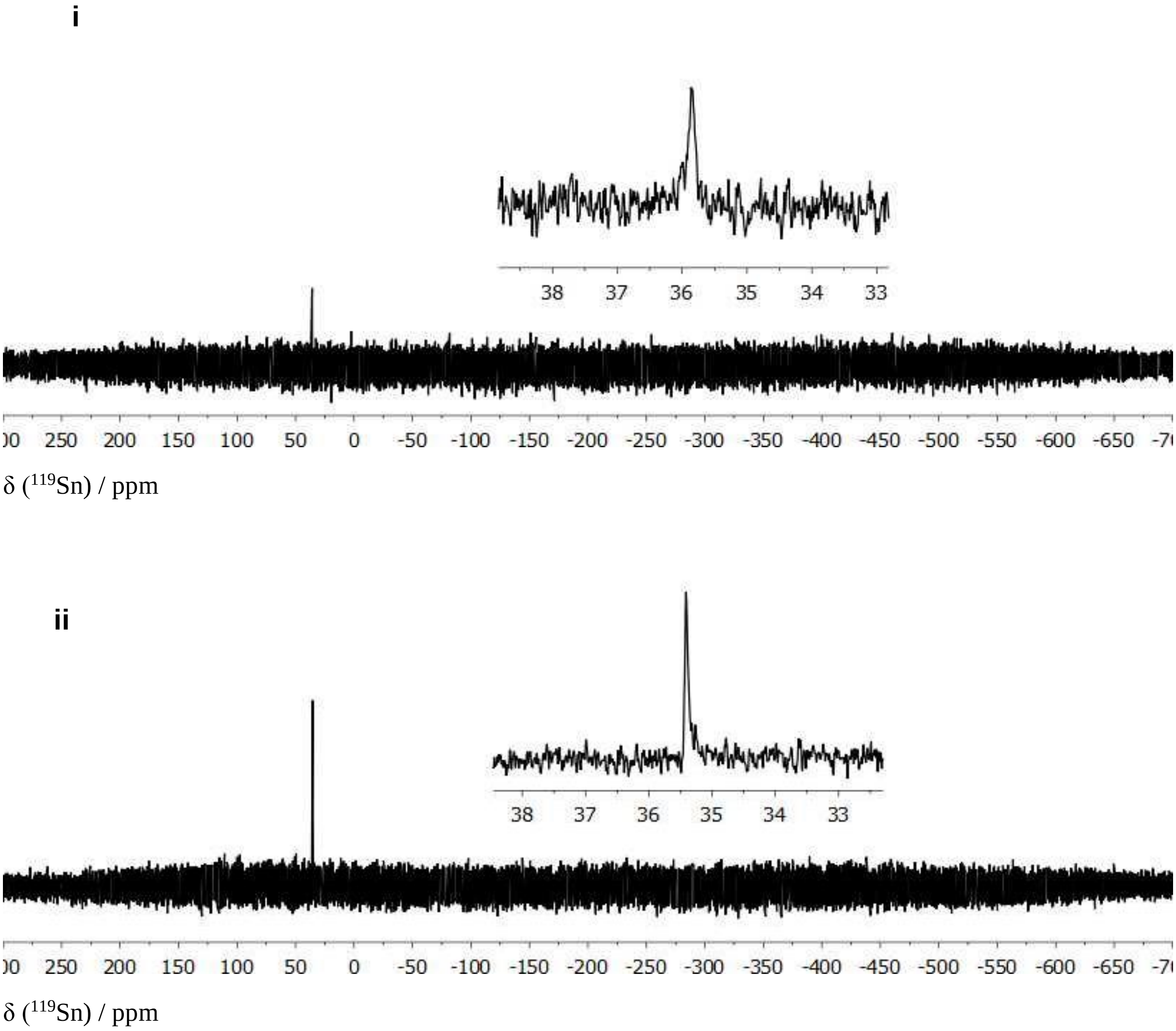

Figure S22b: $^{119}Sn\{^1H\}$ NMR spectra for mixtures of **1**, isopropylamine and H_2O in DCB. **i** is of a ratio of (1:1:5); **ii** is for (1:1:10).

6. Procedure and NMR spectra for the scaled-up reductive amination catalysed of PhCHO and $PhNH_2$ catalysed by 1

A solution of **1** (99.3 mg, 0.25 mmol) in 1,2-difluorobenzene (35 mL) was prepared in a 100 mL Parr 5500 high pressure compact laboratory reactor. The reactor was sealed and sparged with N_2 for 5 minutes, then pressurised with nitrogen (10 bar) and stirred for a further 5 minutes. The reactor was depressurised, and aniline (0.228 mL, 2.50 mmol), benzaldehyde (0.254 mL, 2.50 mmol) and 2,4,6-collidine (33.0 µL, 0.25 mmol) were injected. The reactor was pressurised with hydrogen (35.0 bar, which equates to 50 bar at 150 °C) and heated to 150 °C whilst stirring at 200 rpm. Upon completion of the reaction, the stirrer was stopped, whereupon the reactor was cooled to room temperature and depressurised.

The solvent was removed under reduced pressure, resulting in a dark brown oil. The product was extracted into pentane (10 mL), where it was recrystallised by cooling to -20 °C to obtain **3a** as an off-white crystalline solid (343 mg, 1.87 mmol, 75%).

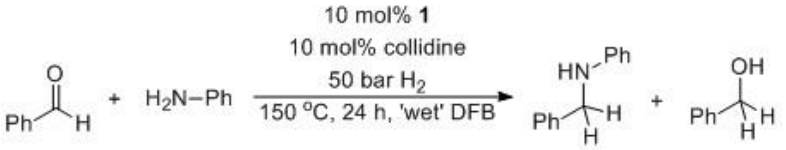

3a **i**

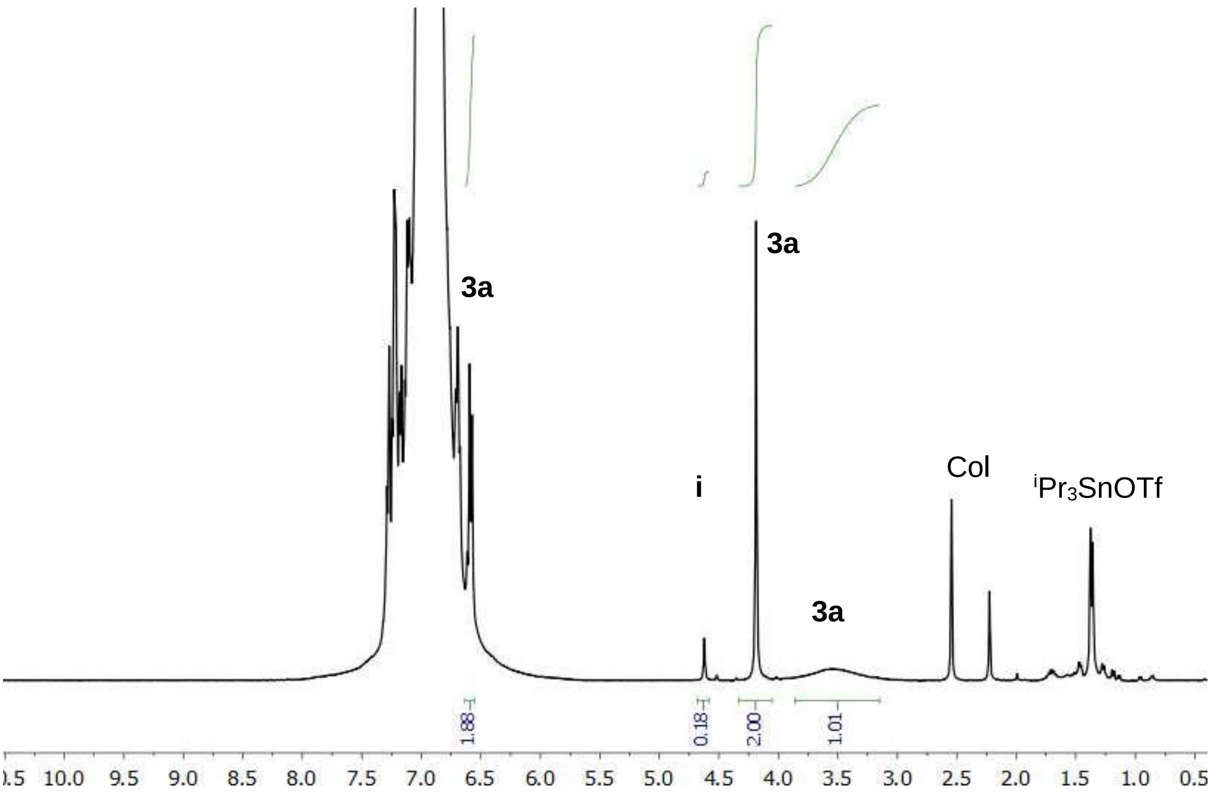

δ (^{1}H) / ppm

Figure S23a: ^{1}H NMR spectrum of the product mixture in DFB before work-up. Conversion (%): **3a** (92%), **i** (8%). Presence of products confirmed by comparison to spectral data of pure, authentic compounds: **3a**,[4] **i**.[2]

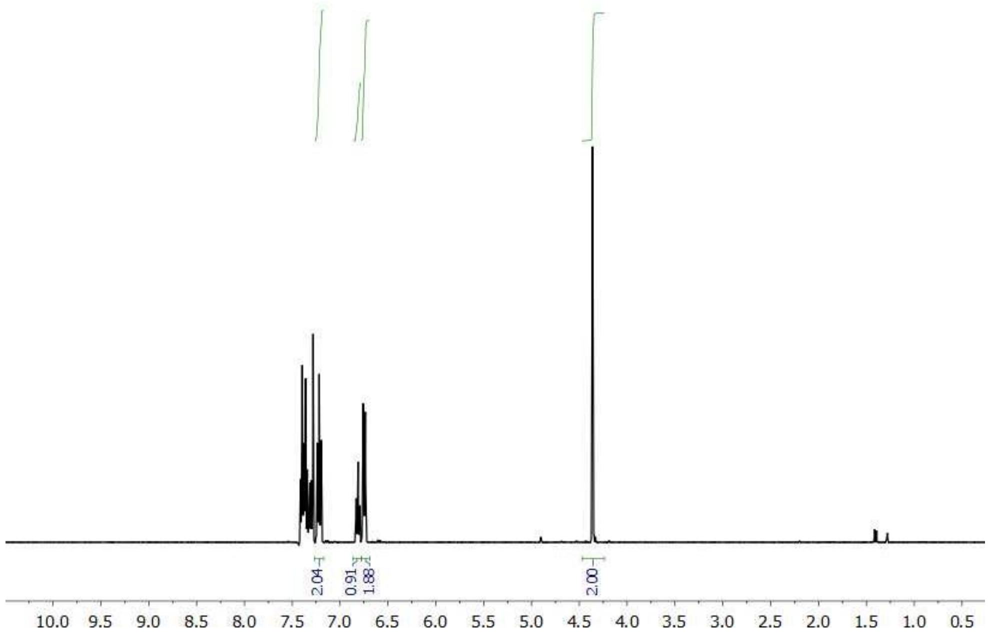

δ (^{1}H) / ppm

Figure S23b: ^{1}H NMR spectrum of purified **3a** verified by comparison to spectral data of the pure, authentic compound in $CDCl_3$.[4]

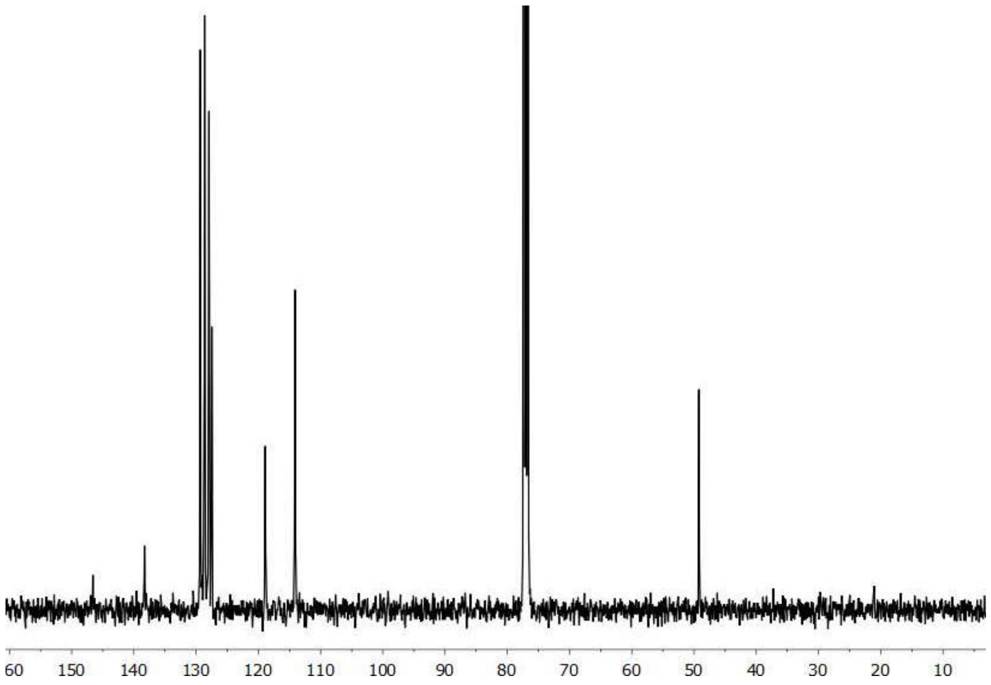

δ (^{13}C) / ppm

Figure S23c: $^{13}C\{^{1}H\}$ NMR spectrum of purified **3a** verified by comparison to spectral data of the pure, authentic compound in $CDCl_3$.[25]

3a: ^{1}H NMR (400 MHz, $CDCl_3$): δ = 7.42-7.34 (m, 6H, Ar–*H*), 7.22 (t, $^{3}J_{HH}$ = 7.3 Hz, 1H, Ar–*H*), 6.81 (t, $^{3}J_{HH}$ = 7.4 Hz, 1H, Ar–*H*), 6.75 (d, $^{3}J_{HH}$ = 7.6 Hz, 2H, Ar–*H*), 4.36 (s, 2H, N–CH_2–Ph). $^{13}C\{^{1}H\}$ NMR (101 MHz, $CDCl_3$): δ = 146.7, 138.3, 129.3, 128.7, 127.9, 127.5, 118.9, 114.1, 49.2.

7. References

[1] D. J. Scott, N. A. Phillips, J. S. Sapsford, A. C. Deacy, M. J. Fuchter, A. E. Ashley, *Angew. Chem. Int. Ed.*, **2016**, *55*, 14738–14742.

[2] G. Zhang, B. L. Scott, S. K. Hanson, *Angew. Chem. Int. Ed.*, **2012**, *51*, 12102–12106.

[3] P.-Q. Huang, Q.-W. Lang, Y.-R. Wang, *J. Org. Chem.*, **2016**, *81*, 4235–4243.

[4] B. S. Takale, S. M. Tao, X. Q. Yu, X. J. Feng, T. Jin, M. Bao, Y. Yamamoto, *Org. Lett.*, **2014**, *16*, 2558–2561.

[5] R. Bisht, B. Chattopadhyay, *J. Am. Chem. Soc.*, **2016**, *138*, 84–87.

[6] A. M. Berman, J. S. Johnson, *J. Am. Chem. Soc.*, **2004**, *126*, 5680–5681.

[7] D. K. T. Yadav, B. M. Bhanage, *Synlett*, **2014**, *25*, 1611–1615.

[8] X. Li, S. Li, Q. Li, X. Dong, Y. Li, X. Yu, Q. Xu, *Tetrahedron*, **2016**, *72*, 264–272.

[9] K. Zhu, M. P. Shaver, S. P. Thomas, *Eur. J. Org. Chem.*, **2015**, *2015*, 2119–2123.

[10] L. Jiang, L. Jin, H. Tian, X. Yuan, X. Yu, Q. Xu, *Chem. Commun.*, **2011**, *47*, 10833.

[11] T. Schwob, R. Kempe, *Angew. Chem. Int. Ed.*, **2016**, *55*, 15175–15179.

[12] P. Liu, R. Liang, L. Lu, Z. Yu, F. Li, *J. Org. Chem.*, **2017**, *82*, 1943–1950.

[13] S. L. Zultanski, J. Zhao, S. S. Stahl, *J. Am. Chem. Soc.*, **2016**, *138*, 6416–6419.

[14] N. Yoshikai, A. Mieczkowski, A. Matsumoto, L. Ilies, E. Nakamura, *J. Am. Chem. Soc.*, **2010**, *132*, 5568–5569.

[15] S. Zhao, C. Liu, G. Yong, J.-C. Xiao, Q.-Y. Chen, *J. Org. Chem.*, **2014**, *79*, 8826–8931.

[16] W. Xie, M. Zhao, C. Chunming, *Organometallics*, **2013**, *32*, 7440–7444.

[17] S. Chakraborty, G. Leitus, D. Milstein, *Angew. Chem. Int. Ed.*, **2017**, *56*, 2074–2078.

[18] K. M. Miyamoto, M. Hoque, S. Ogasa, *J. Org. Chem.*, **2012**, *77*, 8317–8320.

[19] V. Fasano, M. J. Ingleson, *Chem. Eur. J.*, **2017**, *23*, 2217–2224.

[20] G. Jiang, J. Chen, J.-S. Huang, C.-M. Che, *Org. Lett.*, **2009**, *11*, 4568–4571.

[21] R. L. Marshall, I. W. Muderawan, D. J. Young, *J. Chem. Soc. Perkin Trans. 2*, **2000**, 957–962.

[22] A. H. G. Siebum, W. S. Woo, J. Lugtenburg, *Eur. J. Org. Chem.*, **2003**, 4664–4678.

[23] N. Z. Yagafarov, P. N. Kolesnikov, D. L. Usanov, V. V Novikov, Y. V Nelyubina, D. Chusov, *Chem. Commun.*, **2016**, *52*, 1397–1400.

[24] J. Yang, *Dalton Trans.*, **2017**, *46*, 5003–5007.

[25] A. Bartoszewicz, R. Marcos, S. Sahoo, A. K. Inge, X. Zou, B. Martín-Matute, *Chem. Eur. J.*, **2012**, *18*, 14510–14519.

[26] O. Daugulis, M. Brookhart, *Organometallics*, **2002**, *21*, 5926–5934.

[27] H. Fukui, Y. Fukushi, S. Tahara, *Tetrahedron Lett.*, **2005**, *46*, 5089–5093.

[28] J. Barluenga, A. Jiménez-Aquino, F. Aznar, C. Valdés, *J. Am. Chem. Soc.*, **2009**, *131*, 4031–4041.

[29] J. C. Vantourout, R. P. Law, A. Isidro-Llobet, S. J. Atkinson, A. J. B. Watson, *J. Org. Chem.*, **2016**, *81*, 3942–3950.

[30] G. R. Fulmer, A. J. M. Miller, N. H. Sherden, H. E. Gottlieb, A. Nudelman, B. M. Stoltz, J. E. Bercaw, K. I. Goldberg, *Organometallics*, **2010**, *29*, 2176–2179.

[31] D. T. Hog, Oestreich, *Eur. J. Org. Chem.*, **2009**, 5047–5056.

[32] S. R. Roy, S. C. Sau, S. W. Mandal, *J. Org. Chem.*, **2014**, *79*, 9150–9160.

[33] K. H. Lee, B. Lee, K. R. Lee, M. H. Yi, N. H. Hur, *Chem. Commun.*, **2012**, *48*, 4414–4416.

[34] H.-J. Pan, T. W. Ng, Y. Zhao, *Org. Biomol. Chem.*, **2016**, *14*, 5490–5493.
